# Supplementary material for: Design, Development, and Optimisation of Smart Linker Chemistry for Targeted Colonic Delivery—In Vitro Evaluation
Source: Pharmaceutics. 2023 Jan 16;15(1):303. doi: 10.3390/pharmaceutics15010303 (PMC9860859; doi:10.3390/pharmaceutics15010303)
Supplement: Supplementary file 1 [file pharmaceutics-15-00303-s001.zip › pharmaceutics-2143084-supplementary.pdf]

Supporting Information

Design, Development, and Optimisation of Smart Linker Chemistry for Targeted Colonic Delivery—In Vitro Evaluation

Heba S. Abd-Ellah [1,2], Ramesh Mudududdla [1,*], Glen P. Carter [3] and Jonathan B. Baell [4,5,*]

[1] Department of Medicinal Chemistry, Monash Institute of Pharmaceutical Sciences, Parkville, VI 3052, Australia
[2] Department of Medicinal Chemistry, Faculty of Pharmacy, Minia University, Minia 61519, Egypt
[3] Department of Microbiology and Immunology, The University of Melbourne at the Peter Doherty Institute for Infection and Immunity, Parkville, VI 3001, Australia
[4] School of Pharmaceutical Sciences, Nanjing Tech University, No. 30 South Puzhu Road, Nanjing 211816, China
[5] Institute of Drug Discovery Technology (IDDT), Ningbo University, 818 Fenghua Road, Ningbo 315211, China
* Correspondence: ramesh.mudududdla@monash.edu (R.M.); jonathan.baell@njtech.edu.cn or jbaell29@gmail.com (J.B.B.); Tel.: +86-61470330093 (R.M.); +86-61402194571 (J.B.B.)

Table of content

	Figures	Page
Spectrofluorometric assays of probes **13** and **16**	S1-S7	2
Procedure for the synthesis of negative controls (**27** and **29**)	------	10
^{1}H NMR and ^{13}C NMR of negative controls (**27** and **29**)	S8-S13	12
Development of HPLC methods for the assay of Prodrugs **20** and **21**	S14-S16	15
NMR spectra of all synthesised compounds and HRMS are provided for final compounds	S17-S81	19
Proposed mechanism for side-product **24** formation	-------	49

1. Stability of probe 13 at pH 7.5 and 6.5 using the spectrofluorometric assay.

Principal: CTG's fluorescence is reversibly quenched or shifted to another wavelength when the phenol group is conjugated to the pH-sensitive linkers. Upon cyclization of the linker and the release of the free phenol, the fluorescence will be restored (on-mode). Fluorescence can be measured to follow the release of the payload, which is the fluorophore itself.

The optical properties of the free fluorophore were measured in 50% DMSO/buffer at pHs 7.5, and 6.5. CTG gave a strong absorbance at 490 – 516 nm at both pHs with a maximum peak at 510 nm, as shown in **Figure S1 (A)**. Also, it emits strongly between 515 – 560 nm after excitation at 500 nm at pHs 7.5 and 6.5, as shown in **Figure S1 (B, C)**. Since the fluorescence-based assay depends on the difference in fluorescence properties between the free fluorophore and its conjugates, the excitation wavelength must be carefully chosen. The ideal wavelength for exciting the free fluorophore and its conjugates must meet certain criteria. First, the fluorophore should be excited only at this wavelength without great excitation of the fluorophore conjugates. Secondly, the wavelength that gave a reasonable absorbance rather than maximum absorbance should be chosen to avoid exceeding the detector limits. Finally, a long distance between the excitation and emission wavelengths is preferable to minimize the background fluorescence. Accordingly, the *in vitro* tests were conducted by measuring the fluorescence at 555 nm after excitation of the probes at 500 nm, as this wavelength achieved the previous requirements.

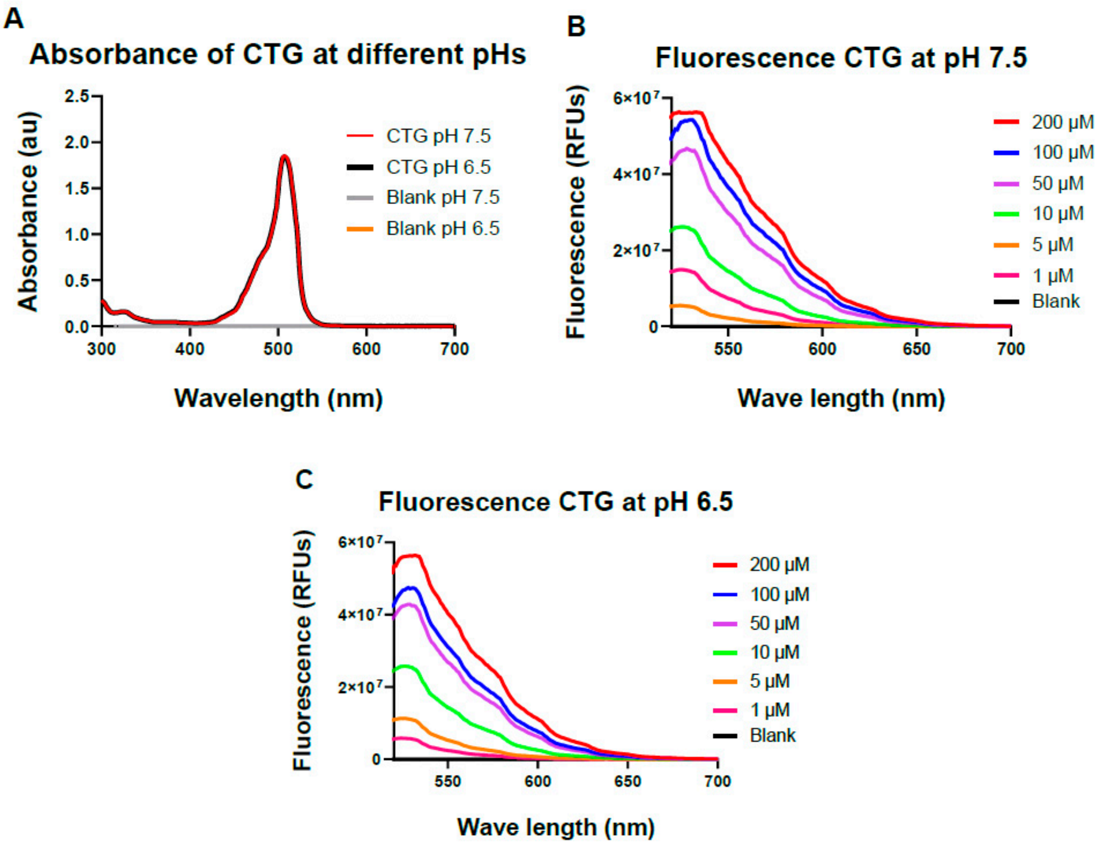

Figure S1. (A) Absorbance spectra of CTG (100 μM) in PBS/DMSO (1:1 v/v, pH= 7.5, 37 °C), PB/DMSO (1:1 v/v, pH= 6.5, 37 °C); Fluorescence spectra of CTG at 555 nm after excitation at 500 nm in **(B)** PBS /DMSO (1:1 v/v, pH = 7.5, 37 °C), **(C)** PB/DMSO (1:1 v/v, pH = 6.5, 37 °C) at different concentrations.

Then, the optical properties of probe **13** were measured in DMSO**.** Probe **13** exhibited a maximum absorbance peak at 457 nm, and the absorbance started to drop off at 500 nm, as shown in **Figure S2 (A)**, therefore 500 nm was the best wavelength to use for excitation. Probe **13** was then excited at 500 nm, and the fluorescence spectra were recorded at different concentrations (200 – 10 μM). The results showed that probe **13** demonstrated considerable fluorescence quenching at all the concentrations tested, as presented in **Figure S2 (B)**.

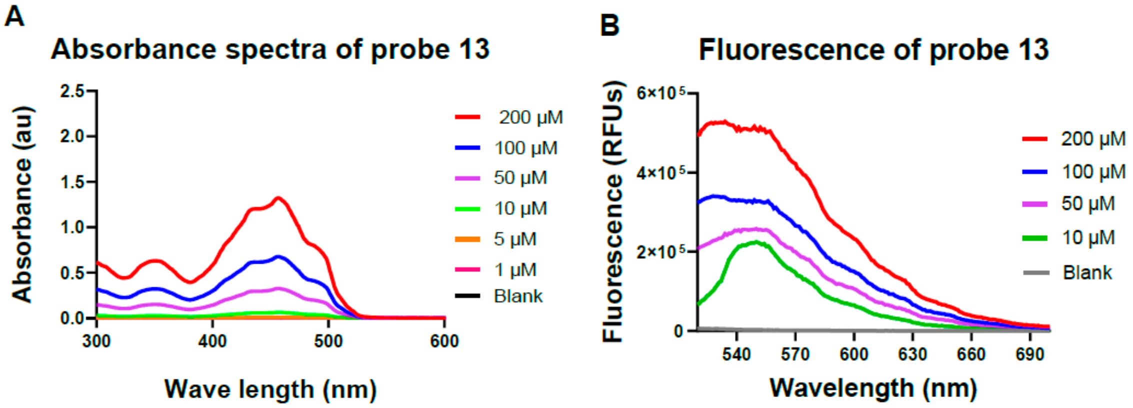

Figure S2. (A) Absorbance spectra of probe **13** in DMSO at different concentrations (37 ºC); (B) Fluorescence spectra of probe **13** in DMSO at different concentrations (37 ºC) (ex: 500 nm, em: 555 nm).

Since the sensing properties of probe **13** were measured in DMSO and compared to the optical properties of CTG that dissolved in buffer/DMSO solution, it was deemed inappropriate to judge based on that comparison. Therefore, a negative control was required to simulate the probe's optical properties in buffer solution without being cleaved to determine whether this was accurate enough. CTG-CH_3 **(27)** was synthesised to use as a negative control as described in **Scheme S1**. Then, the absorbance spectra were taken from CTG-CH_3 after dissolving in 50% DMSO/PBS with a pH of 7.5, revealing a shift in absorbance from 510 nm to a new maximum peak at 457 nm, as shown in **Figure S3 (A).** The absorbance of CTG-CH_3 in 50% PBS/DMSO was similar to the absorbance of probe **13** in DMSO. Following this, CTG-CH_3 was excited at 500 nm after dissolving in 50% DMSO/buffer solutions of pHs 7.5, and 6.5. Results showed a significant decrease in the fluorescence of the negative control compared to CTG of a similar concentration, as shown in **Figure S3 (B)**. These results indicated that the quenching effect was real, and alkylating the fluorophore at the phenol position turned off the fluorescence.

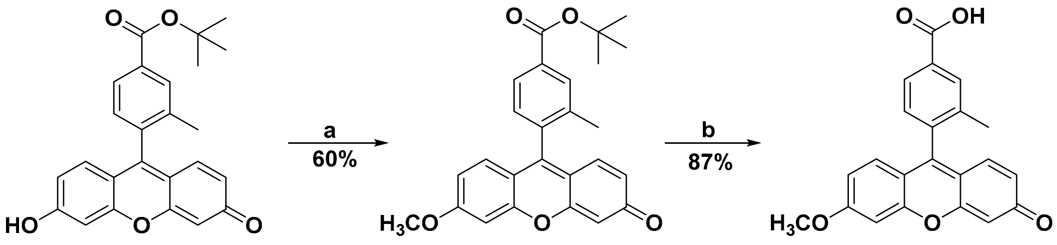

25 26 27

Scheme S1. (a) (i) Cs_2CO_3, DMF, 0 °C, 1 h; (ii) iodomethane, rt, 2h; (b) TFA/dichloromethane, rt, 1 h.

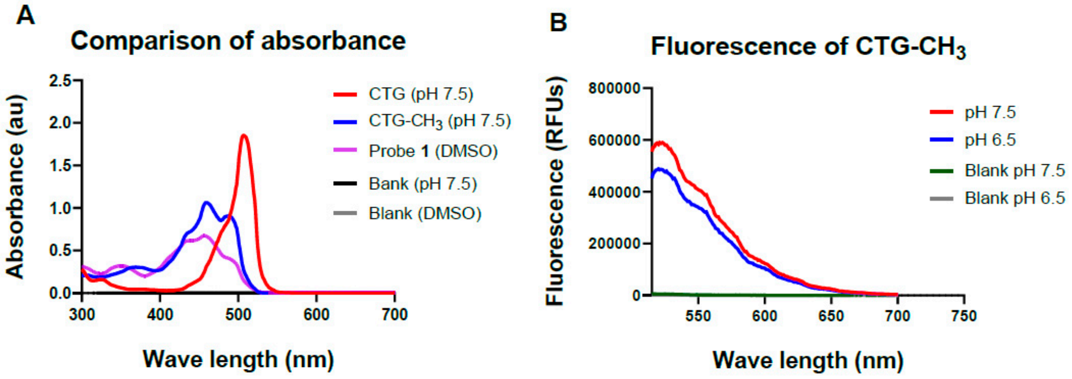

Figure S3. (A) Absorbance spectra of 100 μM of CTG, CTG-CH_3 in PBS /DMSO (1:1 v/v, pH = 7.5, 37 °C), probes **13** in DMSO, **(B)** Fluorescence spectra of CTG-CH_3 (100 μM) in PBS /DMSO (1:1 v/v, pH = 7.5, 37 °C), PB/DMSO (1:1 v/v, pH = 6.5, 37 °C).

Method: Probe **13** was dissolved in DMSO to make a stock solution of 200 μM concentration. Then, 20 nM concentration was prepared by serial dilution method. From this solution, 100 μL was taken and then added to a black-walled, 96-well microplate with each well containing 100 μL of the respective buffer solution to give a final concentration of 10 nM. Similarly, CTG fluorophore dissolved in 50% PBS/DMSO (pH 7.5), 50% PB/DMSO (pH 6.5), and DMSO and was used as a positive control. CTG-CH_3 **(27)** in DMSO, 50% PBS/DMSO (pH 7.5), and 50% PB/DMSO (pH 6.5) were prepared and used as a negative control. Furthermore, probe **13** was dissolved in DMSO at 10 nM concentration and added as an additional control to assure the stability of the probe in the absence of buffer solutions. The microplate was incubated at 37 ºC, and the fluorescence (em: 555 nm, ex: 500 nm) was recorded at every time point until the full release of CTG was obtained.

Results: The fluorescence of probe **13** was low in DMSO and remained consistent over the analysing period, indicating that this probe was stable in DMSO and any increase in the fluorescence would be due to adding the buffer solution, as shown in **Figure S4**. It is worth noting that the negative control (**27**) had its fluorescence quenched in buffer solutions of pHs 7.5 and 6.5, thereby confirming the switch-off mode of the fluorophore when the phenol group was conjugated, as shown **in Figure S4**. CTG gave a high fluorescence in 50% PBS/DMSO and 50% PB/DMSO at pHs 7.5 and 6.5, respectively. Also, it exhibited a high fluorescence in DMSO but is lower than at pHs 7.5 and 6.5. DMSO is a highly polar solvent with non-bonded electrons and should exhibit basic characters and as reported in the literature, sulfuric and hydrochloric acids are completely dissociated in DMSO. The basic strength of DMSO is comparable to that of H_2O. CTG has a low pK_a (4.33), which permits it to partially dissociates in DMSO, resulting in high fluorescence.

Adding buffer solutions of pHs 7.5 and 6.5 to probe **13** caused an immediate increase in the fluorescence intensity that reached the same fluorescence level as the positive control (CTG) after 3 min. We believed that probe **13** cyclizes immediately due to the short distance between the amine and the carbonate group, allowing five-membered ring formation. The CTG release from probe **13** was almost the same at both pHs, as shown in **Figure S4 (A, B)**.

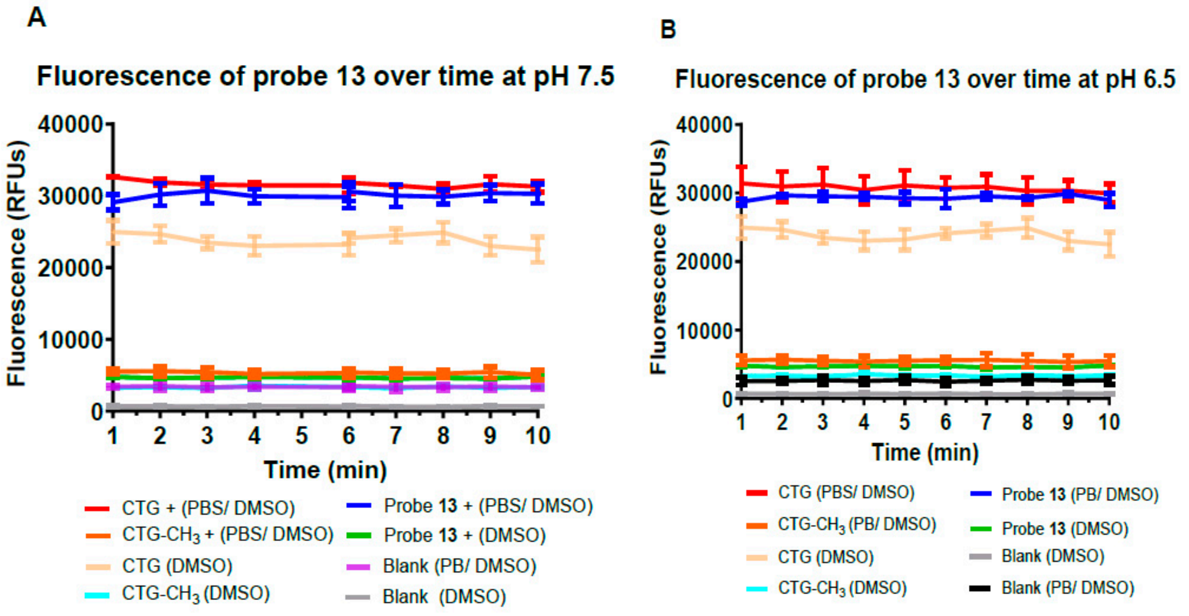

Figure S4. Fluorescence (ex: 500 nm, em: 550 nm) of probe **13** (10 nM) when incubated in **(A)** PBS /DMSO (1:1 v/v, pH = 7.5, 37 °C), **(B)** PB/DMSO (1:1 v/v, pH = 6.5, 37 °C), respectively. Values were recorded in triplicate. Error bars are S.E.M

2. The stability of probe 16 at pHs 7.5 and 6.5 using a fluorometric assay.

The optical properties of DCM-OH were first evaluated at pHs 7.5 and 6.5. Upon dissolving DCM-OH in the 50% DMSO/buffer, purple colour was immediately formed which was recognizable by the naked eye. DCM-OH exhibited one prominent band in the visible region at 564 nm, as shown in **Figure S5 (A)**. However, there was ~7-fold loss in the DCM-OH absorbance at pH 6.5 at a concentration of 200 μM compared with the absorbance measured at pH 7.5. This due to the small amounts of $DCM\text{-}O^-$ that were likely to be formed at pH 6.5. The pK_a value of DCM-OH is reported to be 7.21 [36], therefore, decreasing the pH will decrease the DCM-OH ionization potential. On the other hand, a new prominent peak appeared at 448 nm at pH 6.5. This was likely due to the unionized form of DCM-OH, as shown in **Figure S5 (A)**. A strong emission band was observed at 687 nm after excitation of DCM-OH at 564 nm at both pHs, as shown in **Figure S5 (B, C)**. Also, there was about a ~3-fold loss in the fluorescence of DCM-OH at pH 6.5 compared with that measured at pH 7.5 at a concentration of 200 μM, as shown in **Figure S5 (B)**.

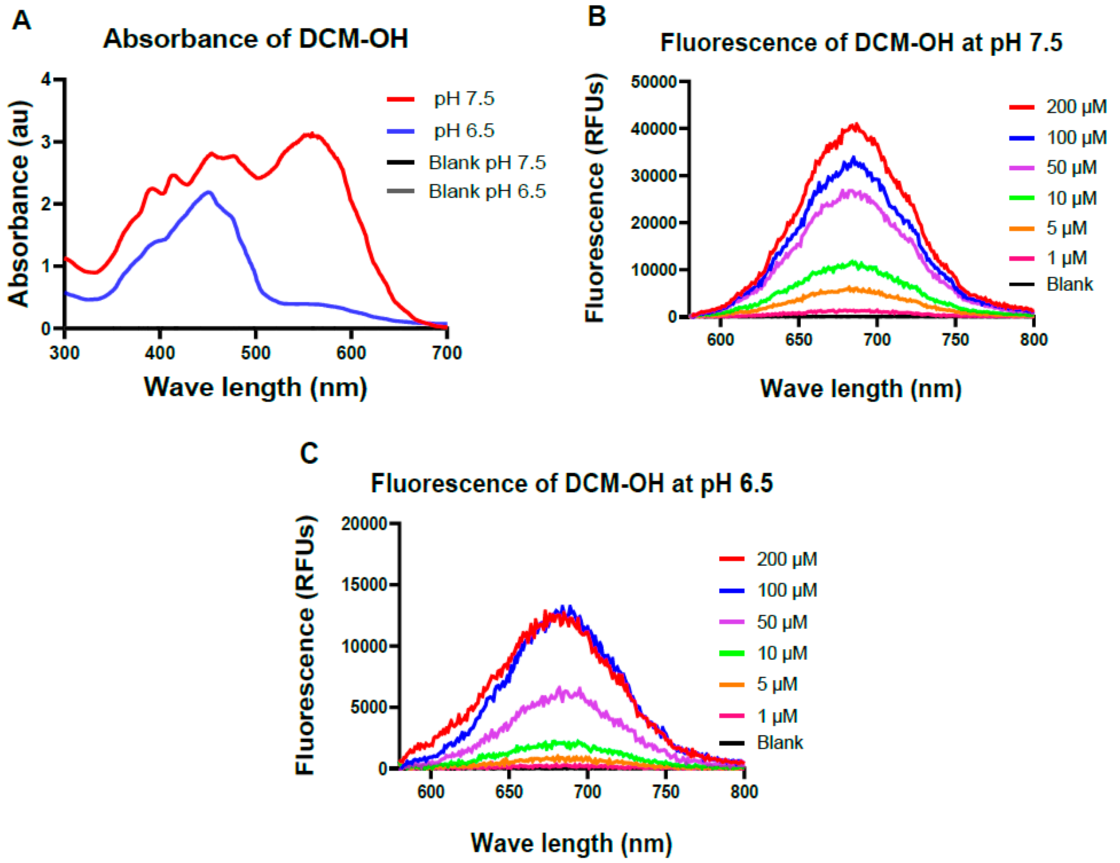

Figure S5. (A) Absorbance of DCM-OH (200 μM) when incubated in PBS/DMSO (1:1 v/v, pH= 7.5, 37 °C), PB/DMSO (1:1 v/v, pH= 6.5, 37 °C); Fluorescence spectra of DCM-OH in (B) PBS/DMSO (1:1 v/v, pH = 7.5, 37°C), **(C)** 50% PB/DMSO (1:1 v/v, pH = 6.5, 37°C) (ex: 564 nm, em: 687 nm).

DCM-CH_3 **(29)** was used as a negative control and has been synthesised via reacting compound **28** with 4-methoxy benzaldehyde as described in **Scheme S2**. Results showed that probe **16** and DCM-CH_3 had no absorbance at 564 nm and another prominent band appeared at 448 nm, **Figure S6 (A, B)**. Also, they showed no fluorescence at 687 nm (<1000 RFUs) after excitation at 564 nm, as shown in **Figure S6 (C)**. This confirms that the conjugation at the phenolic position was able to quench the fluorescence.

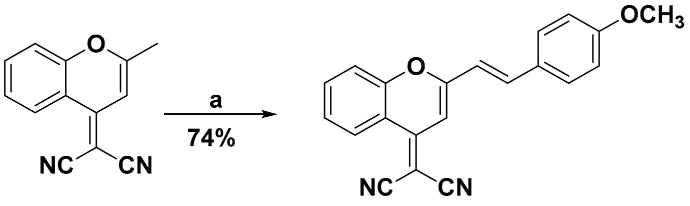

28 **29**

Scheme S2. (a) 4-methoxy benzaldehyde, piperidine, acetonitrile, reflux, 4 h.

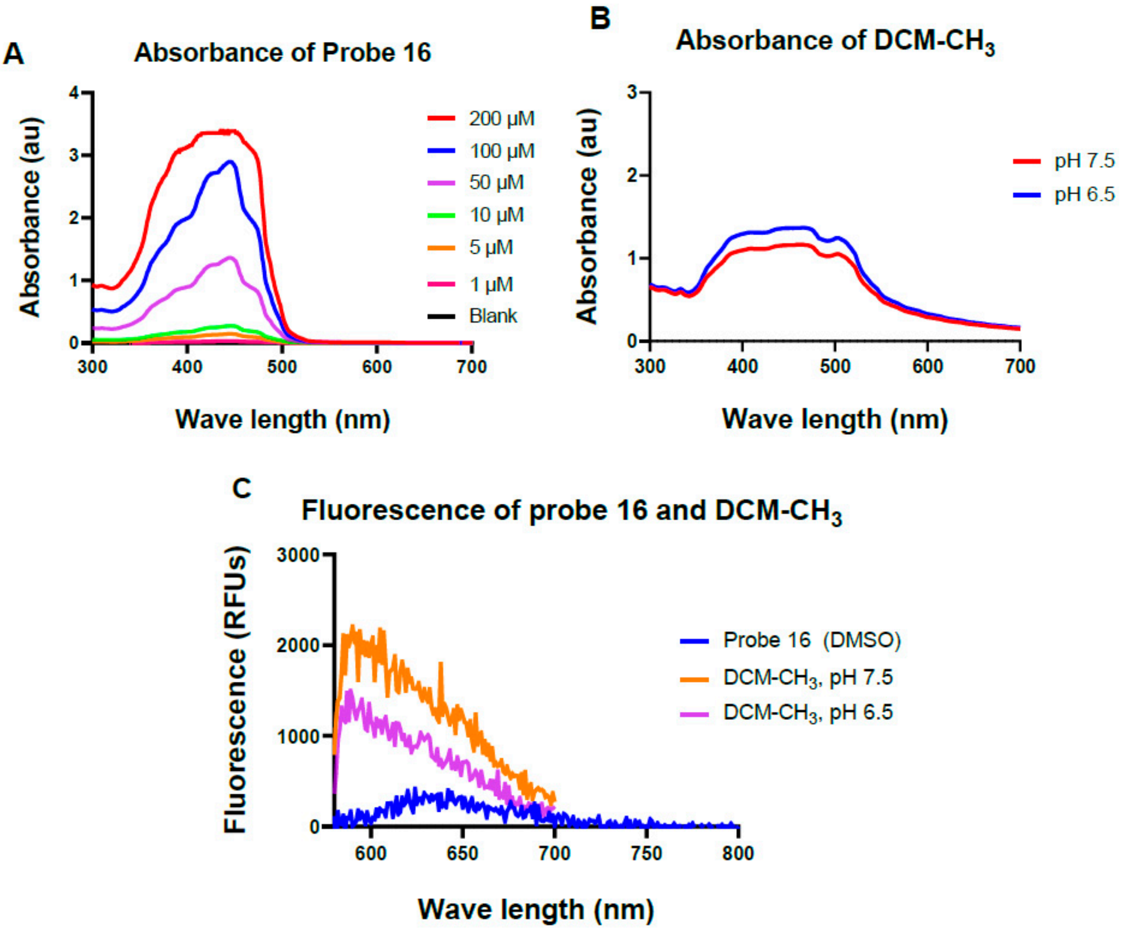

Figure S6. Absorbance spectra of **(A)** probe **16** in DMSO (37 °C), **(B)** DCM-CH_3 when incubated in 50% PBS/DMSO (1:1 v/v, pH = 7.5, 37 °C), 50% PBS/DMSO (1:1 v/v, pH = 6.5, 37 °C), **(C)** Fluorescence spectra of probes **16** in DMSO (37 °C), and DCM-CH_3 when incubated in 50% PBS/DMSO (1:1 v/v, pH = 7.5, 37 °C), PBS/DMSO (1:1 v/v, pH = 6.5, 37 °C) (ex: 564 nm, em: 687 nm).

Method: Stock solution of probe **16** was prepared in DMSO (20 µM) by serial dilution technique and then from this solution, 100 µL was added into 96-well microplate with wells containing 100 µL of PBS/DMSO (pH 7.5) to achieve 10 µM concentration. Similarly, 200 µL of the negative control (DCM-CH_3) as well as the positive control (DCM-OH) were added at 10 µM concentration, then the samples were incubated at 37 °C. The fluorescence was measured at every time point for the tested solutions until the full fluorophore release was achieved.

To study the release of the fluorophore from probe **16** at pH 6.5, 100 µM concentration was used to overcome the decrease in the fluorescence of DCM-OH at this pH and method was the same as pH 7.5.

Results: It was observed that the purple color was formed immediately in the case of probe **16** at pH 7.5 and 6.5. The color change from yellow to purple permits the colorimetric detection of the probe by the naked eye, and was invoked by a 119 nm red shift in the absorption spectrum. The results showed that DCM-OH was released immediately from probe **16** at pH 7.5 and 6.5 with a minor increase over the first 2 min as shown in **Figure S7 (A, C).**

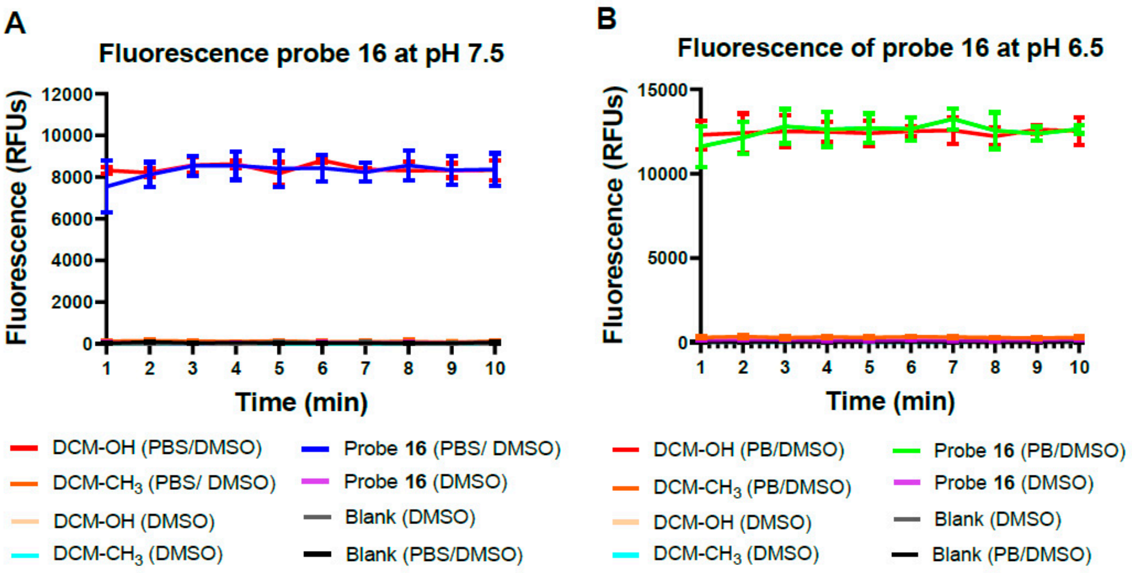

Figure S7. The correlation of fluorescence with respect to time for 10 µM and 100 µM of probe **16** when incubated in **(A)** PBS /DMSO (1:1 v/v, pH = 7.5, 37 °C); (B) PB/DMSO (1:1 v/v, pH = 6.5, 37 °C), respectively (ex: 564 nm, em: 689 nm).

3. Procedure for the synthesis of the negative controls (27 and 29)

***Tert*-butyl 4-(6-methoxy-3-oxo-3*H*-xanthen-9-yl)-3-methylbenzoate (26).** A mixture of compound **25** (0.10 g, 0.25 mmol) and Cs_2CO_3 (0.24 g, 0.75 mmol) in 15 mL DMF was stirred at 0 ºC for 1 h. Then, iodomethane (0.45 g, 0.63 mmol) was added dropwise, and the reaction was monitored by TLC (5% MeOH in dichloromethane). After 2 h, compound **25** was completely consumed, the reaction was quenched with saturated brine, and the crude product was extracted with ethyl acetate. Then, the combined organic layers were washed with water, dried over $MgSO_4$, and concentrated under reduced pressure. The product was purified by column chromatography using 30% EtOAc in pet. spirits, and obtained as an orange solid (62 mg, 60%). ^{1}H NMR (400 MHz, $CDCl_3$): δ 8.03 (s, 1H), 7.99 (dd, *J* = 7.9, 1.1 Hz, 1H), 7.24 (s, 1H), 7.00 (d, *J* = 2.3 Hz, 1H), 6.96 – 6.89 (m, 2H), 6.79 (dd, *J* = 8.9, 2.3 Hz, 1H), 6.67 – 6.56 (m, 2H), 3.95 (s, 3H), 2.12 (s, 3H), 1.65 (s, 9H); ^{13}C NMR (101 MHz, $CDCl_3$): δ 179.2, 165.3, 164.7, 158.9, 154.9, 148.5, 136.9, 136.8, 133.4, 131.6, 130.5, 130.3, 129.4, 129.2, 127.3, 118.3, 114.0, 113.78, 106.2, 100.7, 81.8, 56.2, 28.4, 19.7; LRMS (ESI+) *m/z*: 417.2 $[M+H]^+$; HRMS *m/z* (ESI) calcd. for $C_{26}H_{24}O_5$ $[M+H]^+$ 417.1697, found 417.1702; HPLC: t_R = 6.70 min (> 99%) at 254 nm.

4-(6-Methoxy-3-oxo-3*H*-xanthen-9-yl)-3-methylbenzoic acid (27).
Compound **26** (50 mg, 0.06 mmol) was stirred with 2 mL of TFA/ dichloromethane (1:1) at rt. The reaction was monitored by TLC using 10% MeOH in dichloromethane and when completed it was concentrated under reduced pressure to give the desired product as an orange solid (30 mg, 87%). ^{1}H NMR (400 MHz, DMSO-d_6): δ 8.06 (s, 1H), 7.98 (d, *J* = 7.9 Hz, 1H), 7.44 (d, *J* = 7.9 Hz, 1H), 7.33 (d, *J* = 1.0 Hz, 1H), 7.99 – 6.93 (m, 3H), 6.56 (dd, *J* = 9.6, 1.8 Hz, 1H), 6.43 (d, *J* = 1.8 Hz, 1H), 3.95 (s, 3H), 2.08 (s, 3H); ^{13}C NMR (101 MHz, DMSO-d_6): δ 182.5, 166.9, 165.1, 158.4, 154.6, 150.0, 136.4, 136.4, 132.0, 131.2, 130.6, 129.5, 129.4, 128.8, 127.0, 117.2, 114.5, 113.7, 104.5, 100.8, 56.5, 19.0; LRMS (ESI+) *m/z*: 361.1 $[M+H]^+$; HRMS *m/z* (ESI) calcd. for $C_{22}H_{16}O_5$ $[M+H]^+$ 361.1071, found 361.1073; HPLC: t_R = 5.00 min (>99%) at 254 nm.

***(E)*-2-(2-(4-Methoxystyryl)-4*H*-chromen-4-ylidene)malononitrile (29).**
Compound **29** was synthesised via refluxing compound **28** (0.30 g, 1.44 mmol) and 4-methoxy benzaldehyde (0.21 g, 1.70 mmol) in the presence of piperidine. The pure compound was obtained as a fluffy orange crystal (0.35 g, 74%). ^{1}H NMR (400 MHz, DMSO-d_6) δ 8.74 (dd, *J* = 8.4, 1.4 Hz, 1H), 7.93 (m, 1H), 7.80 (dd, *J* = 8.5, 1.3 Hz, 1H), 7.78 – 7.69 (m, 3H), 7.62 (m, 1H),

7.37 (d, J = 16.1 Hz, 1H), 7.05 (d, J = 8.9 Hz, 2H), 7.00 (s, 1H), 3.83 (s, 3H); ^{13}C NMR (101 MHz, $CDCl_3$): δ 161.9, 158.1, 153.0, 152.5, 138.9, 134.7, 129.8, 127.6, 126.0, 118.7, 118.1, 117.1, 116.5, 116.0, 114.9, 106.4, 55.6; LRMS (ESI+) m/z: 327.0 $[M+H]^+$; HRMS m/z (ESI) calcd. for $C_{21}H_{14}N_2O_2[M+H]^+$ 327.1128, found 327.1122; HPLC: t_R = 7.54 min (> 99%) at 254 nm.

4. NMR spectra of compounds 27 and 29.

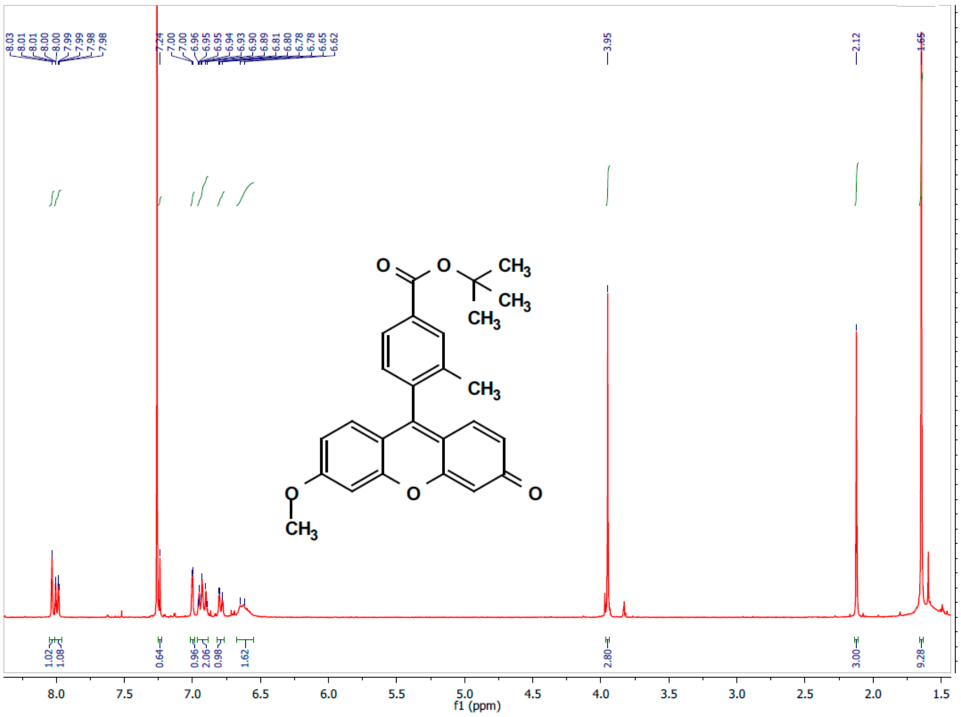

Figure S8. ^{1}H NMR spectrum of **26**.

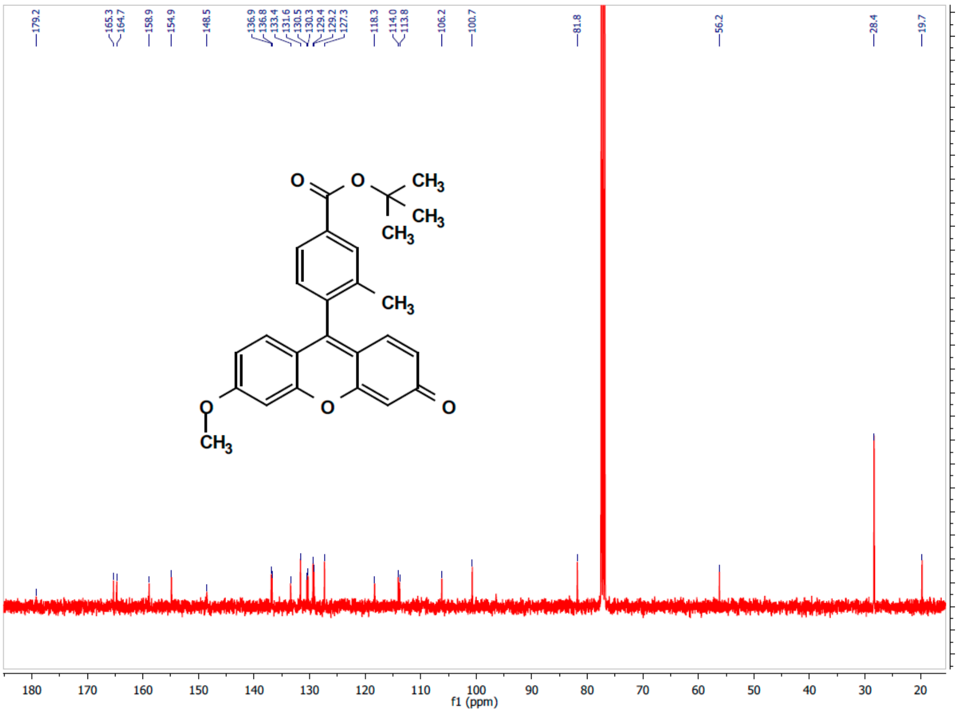

Figure S9. ^{13}C NMR spectrum of **26**.

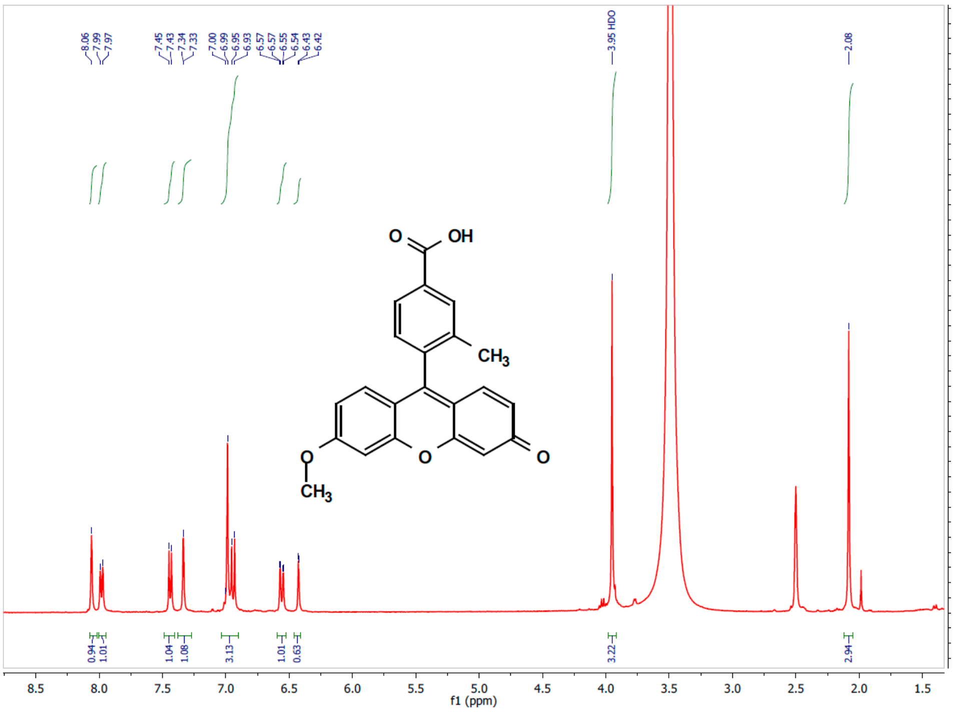

Figure S10. ^{1}H NMR spectrum of **27**.

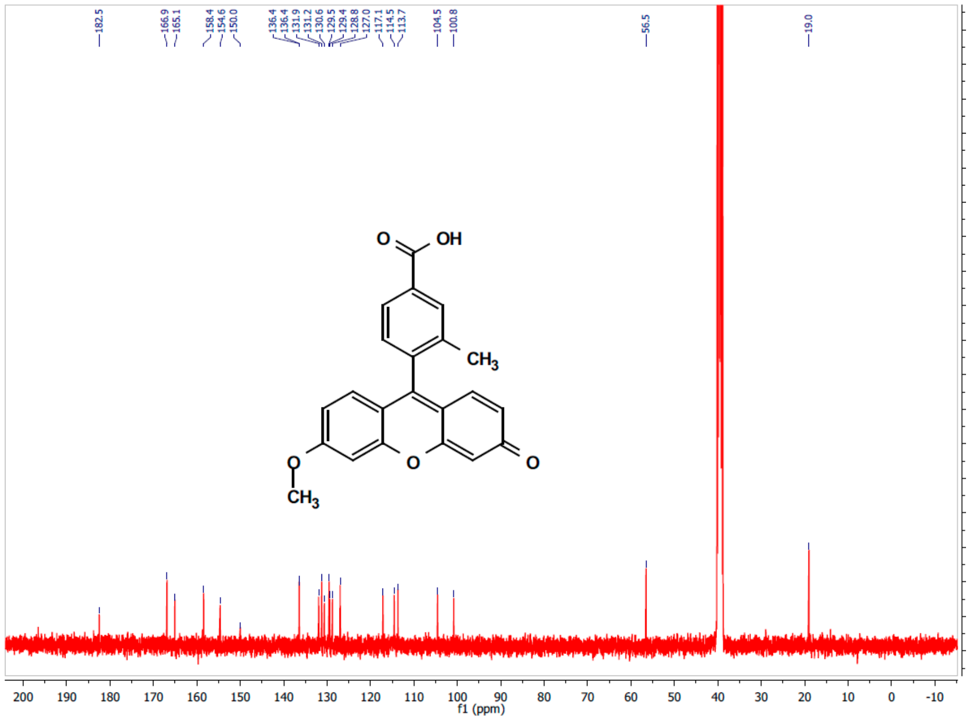

Figure S11. ^{13}C NMR spectrum of **27**.

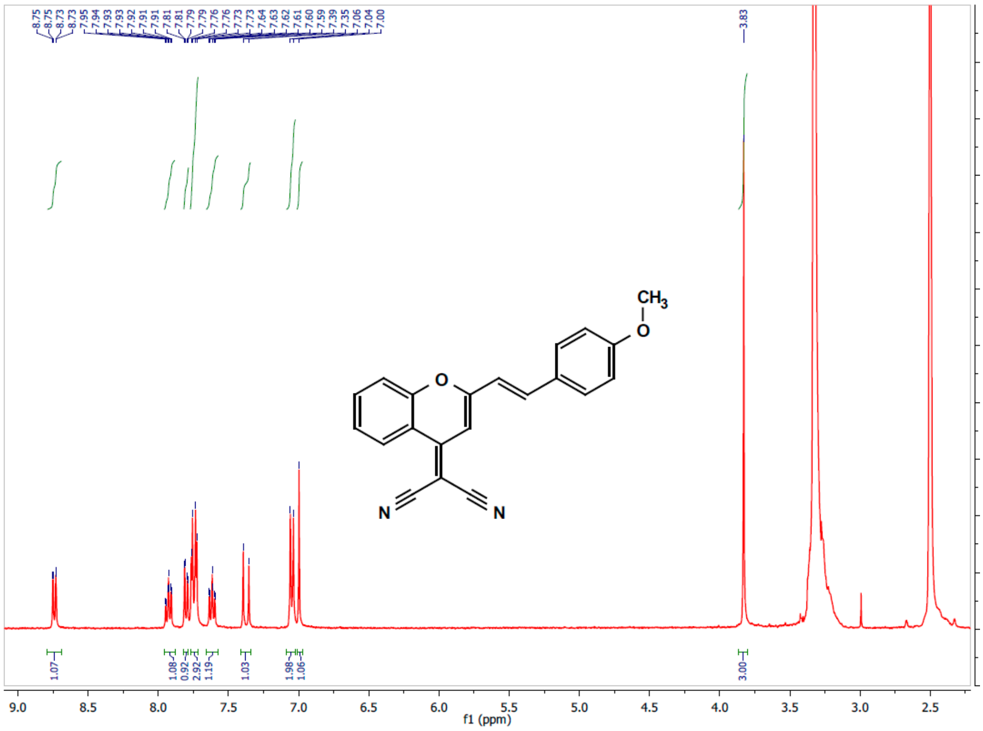

Figure S12. ^{1}H NMR spectrum of **29**.

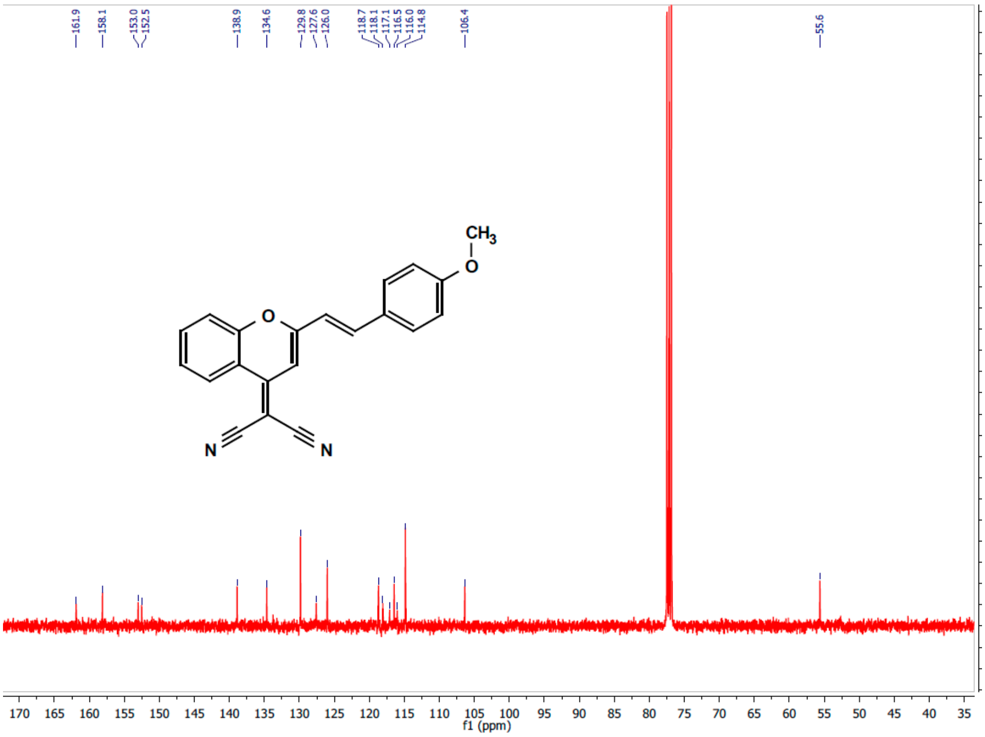

Figure S13. ^{13}C NMR spectrum of **29.**

5. Development of HPLC-based assay to study the stability of Msl prodrugs.

Msl is a polar compound that exhibits amphoteric properties due to the presence of the carboxylic group (–COOH pK_a = 3), the aromatic amino group (–NH_3^+ pK_a = 6), and the phenolic group (–OH pK_a = 13.9) which complicate its extraction, separation, and detection [44]. Literature survey reported several analytical methods to quantify Msl in pure drug, pharmaceutical dosage forms, or biological samples using spectrophotometry [40, 41], HPLC [42, 43], or ultra-performance liquid chromatography (UPLC) [43]. Literature methodologies have been investigated to find a proper HPLC method for determining the amount of Msl released. There were several reported HPLC methods with different mobile phases at different wavelengths. Some of these methods include using methanol/water (1:1) at 220 nm [38], methanol/water (1:1) at 230 nm, mixed phosphate buffer/acetonitrile (65: 35 v/v, pH 6.5) at 258 nm, or phosphate buffer/methanol (60:40 v/v, pH 6.8) at 235 nm [39]. Nevertheless, none of these methods are suitable to use in the current *in vitro* assay with different pH and temperature conditions, and thus a new methodology has to be developed. Initially, the absorbance of Msl was measured at different pHs using a microplate reader to find the ideal

wavelength to run in the assay. Msl was dissolved in ethanol/(KCl/HCl) buffer (1:1, pH 1.2), ethanol/acetate buffer (1:1, pH 5.0) ethanol/PB (1:1, pH 6.5), and ethanol/PB (1:1, pH 7.5) to prepare a 100 µM concentration and then scanned within the wavelength region of 220-400 nm against the blank solution. The results indicated that the blank solution had a high absorbance at 220-300 nm and its absorbance started to decrease after 300 nm and this may be due to the plastic in the 96-well microplate absorbing at the UV region of 220-300 nm. Therefore, it was decided to proceed with the *in vitro* tests by measuring the absorbance at wavelength > 300 nm where there is no interference with the blank.

Msl showed a good absorbance at 300-350 nm at pHs 5.0, 6.5, and 7.5 with a maximum peak at 331 nm while it had no absorbance at 331 nm at pH 1.2. Instead, it had a prominent absorbance band at 304 nm at pH 1.2, as shown in **Figure S14**. These results are consistent with those reported in the literature [41, 11]. A wavelength of 310 nm, at which Msl has a good absorbance at all pHs, was chosen.

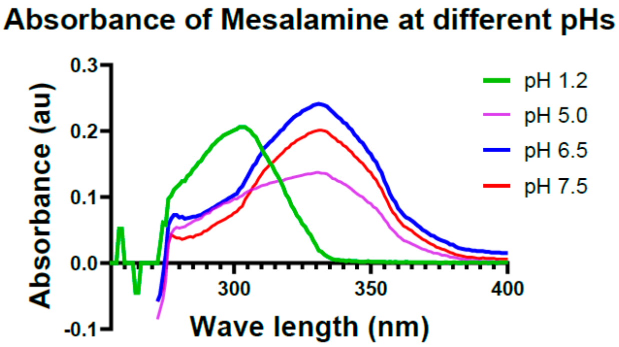

Figure S14. Absorbance spectra of 100 µM of Msl after blank subtracting in PB/ethanol (1:1 v/v, pH = 7.5, 37 °C), PB/ethanol (1:1 v/v, pH = 6.5, 37 °C), acetate buffer/ethanol (1:1 v/v, pH = 5.0, 37 °C), and (KCl/HCl) buffer/ethanol (1:1 v/v, pH = 1.2, 37 °C).

Then, to confirm that it was the appropriate wavelength to select in the HPLC method, 100 µM of Msl was injected in reversed-phase HPLC and was eluted via a gradient elution using water/acetonitrile (5-100%) as the mobile phase in the presence of 0.1% of TFA as additive. The flow rate was kept at 1.0 ml/min and the eluent was scanned using a photodiode array detector at four wavelengths: 220 nm, 230 nm, 254 nm, and 310 nm. The results revealed that Msl eluted at a retention time of 1.19 min with a higher absorbance at 220 nm and 230 nm compared to 310 nm. However, 310 nm was the best wavelength to run in the assay as it is far away from the UV cut-off wavelength of the mobile phase, and the reported value is > 260 nm [23]. The results presented are compatible with the results that reported 0.37 and 0.25 absorbance of 0.1% of TFA in MeCN at wavelengths of 220 nm and 230 nm, respectively [23], as shown in **Figure S15**.

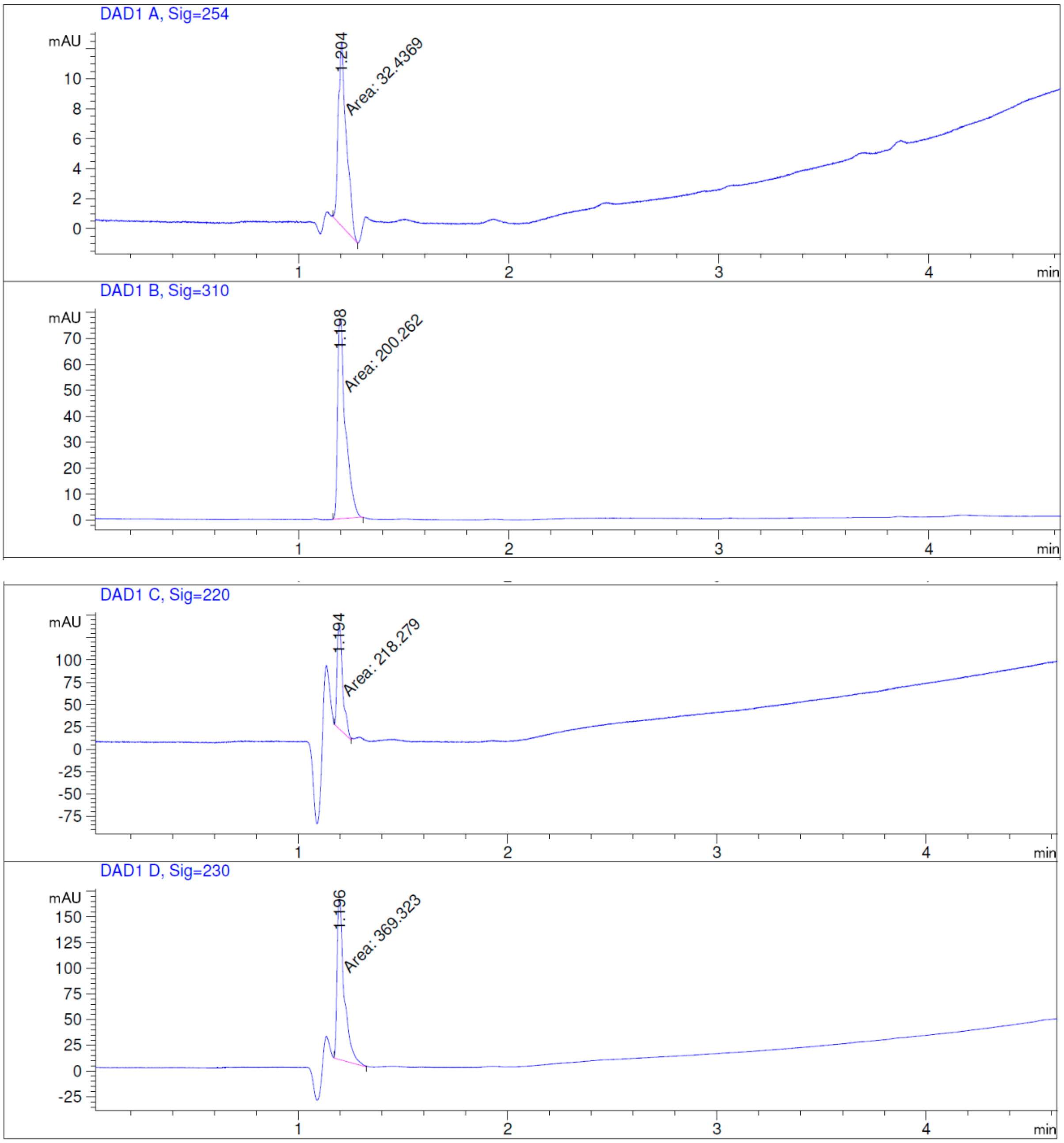

Figure S15. HPLC-ultraviolet photodiode array detector chromatogram of 100 µM of Msl in ethanol/buffer (1:1) at pH 6.5.

After that, calibration curves of Msl were established to estimate the best concentration to be used in the *in vitro* assays. Initially, a stock solution of Msl (2000 µM) was prepared by dissolving it in ethanol and then diluting with the respective buffer to obtain different concentrations: 1000 µM, 500 µM, 250 µM, 125 µM, 100 µM, 62.5 µM, 50 µM at different pHs. Samples were then injected into HPLC under the previously mentioned conditions and calibration curves were obtained by plotting the peak areas against their corresponding concentrations. The regression equations for Msl at pHs of 7.5 and pH 6.5 were calculated to be y= 1.691x-8.387 and y= 1.447x-4.674, respectively. Furthermore, the correlation coefficients (R^2) were found to be 0.999, thus confirming the linearity of the used method under the

specified measured concentrations, as shown in **Figure S16 (A, B)**. It was decided to use a concentration of 250 µM as it gave a good instrument response at the selected wavelength.

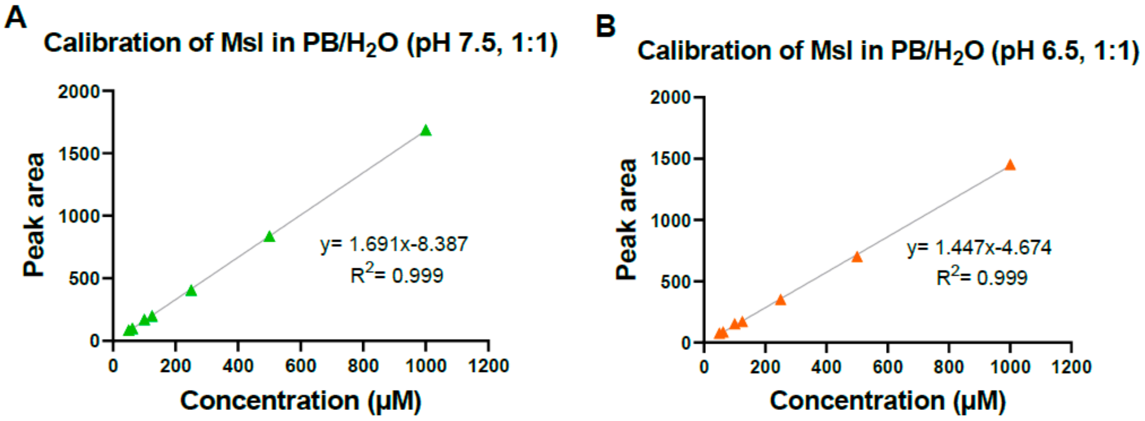

Figure S16. Calibration curves of Msl when incubated in (**A**) PB/H_2O (1:1 v/v, pH = 7.5, 37 °C) and **(B)** PB/H_2O (1:1 v/v, pH = 6.5, 37 °C).

6. NMR spectra of all synthesised compounds .

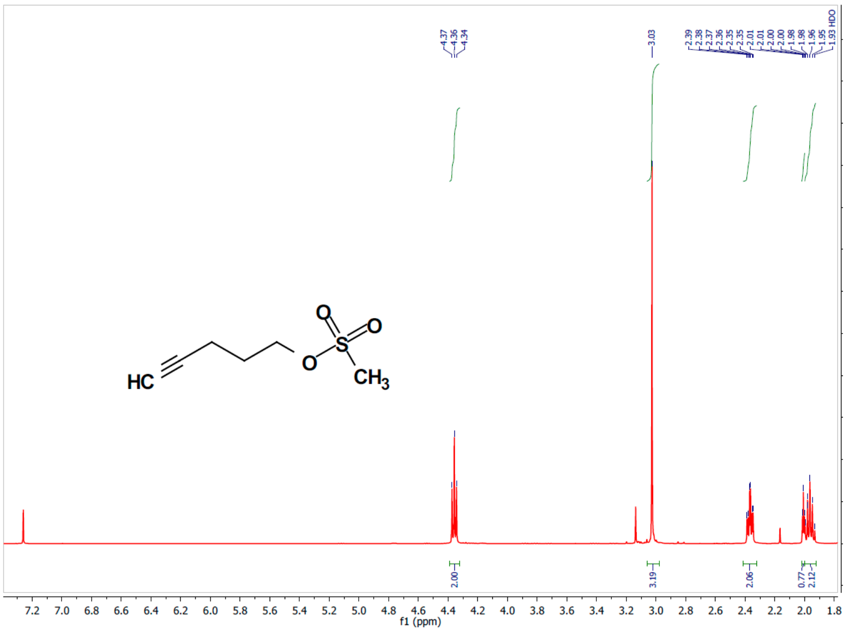

Figure **S17**. ^{1}H NMR spectrum of 6.

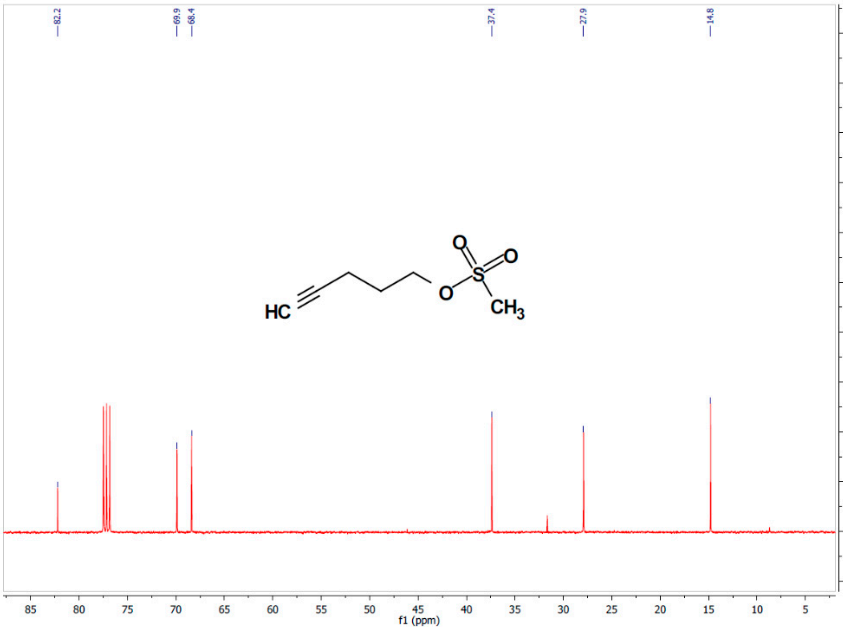

Figure S18. ^{13}C NMR spectrum of 6.

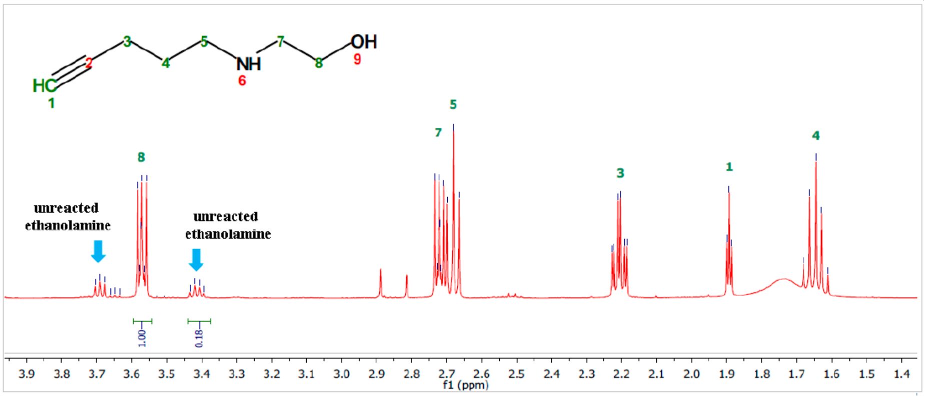

Figure S19. ^{1}H NMR spectrum of **7a**.

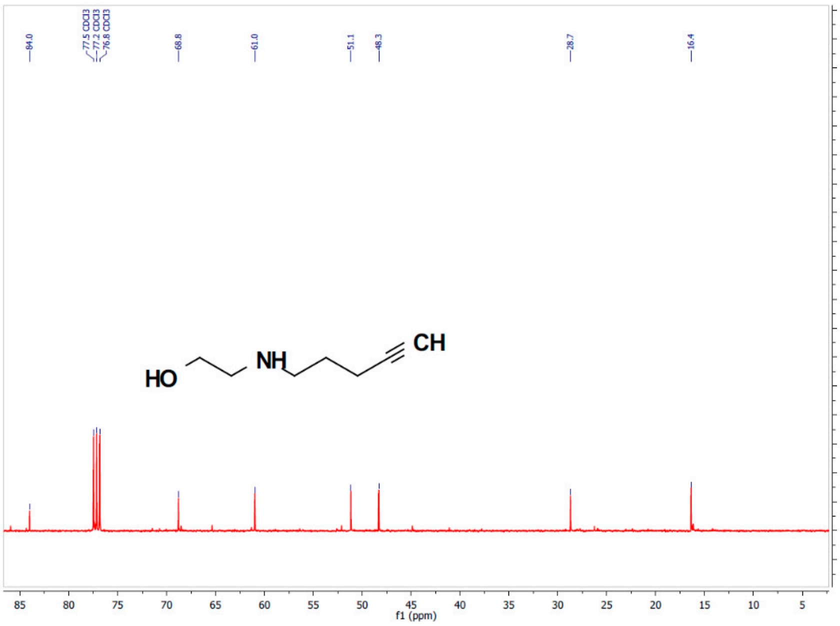

Figure S20. ^{13}C NMR spectrum of **7a**.

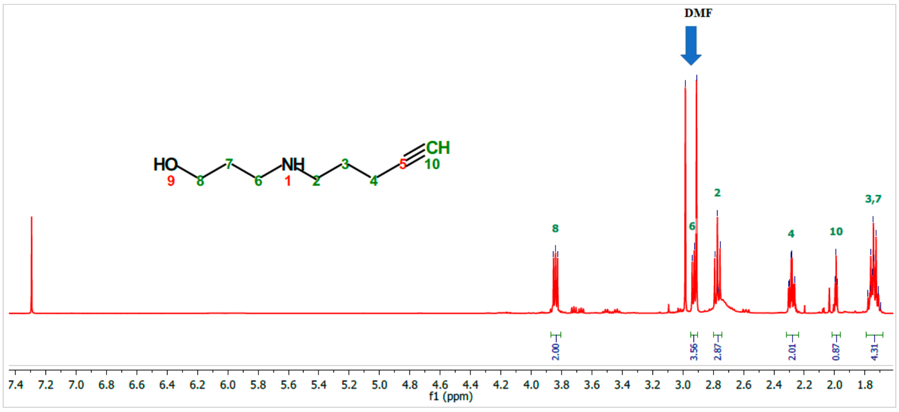

Figure S21. ^{1}H NMR spectrum of **7b**.

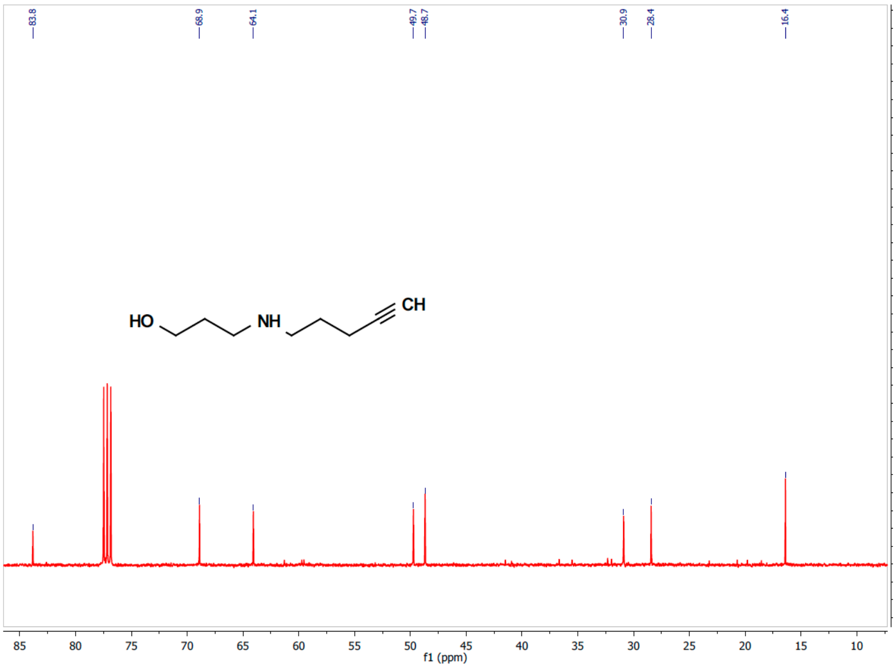

Figure S22. ^{13}C NMR spectrum of **7b**.

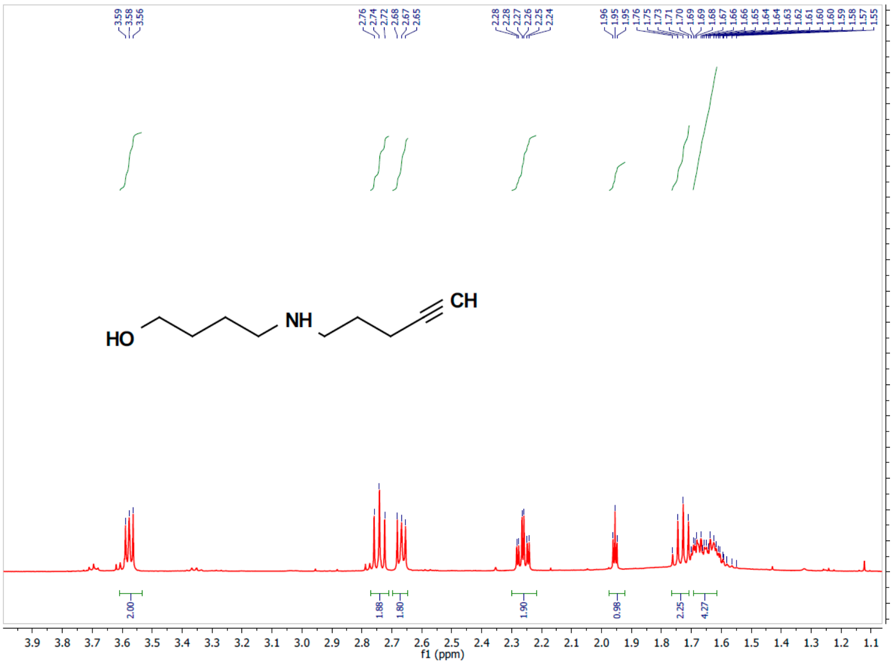

Figure S23. ^{1}H NMR spectrum of **7c.**

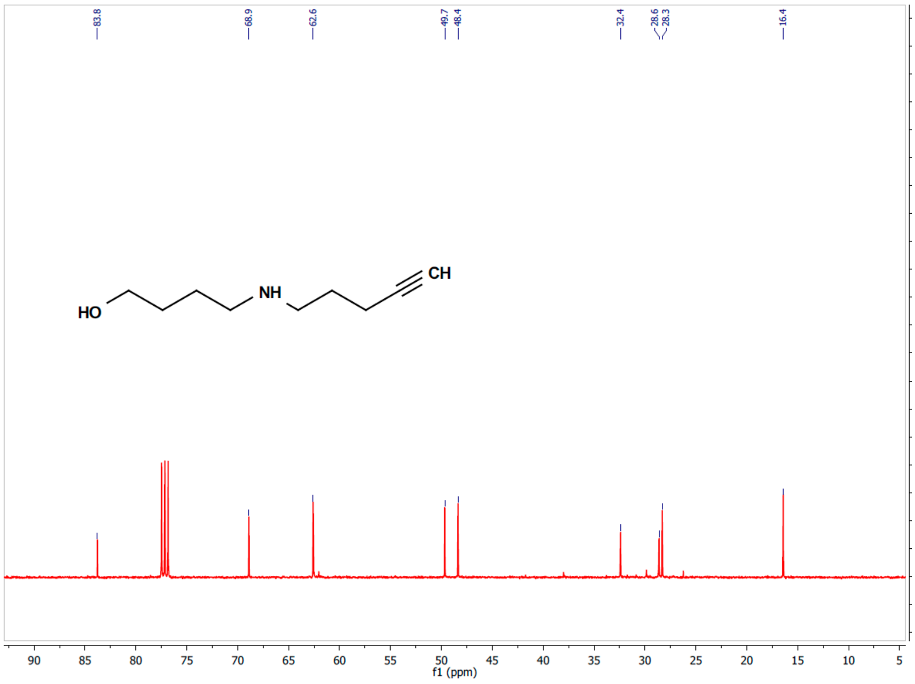

Figure S24. ^{13}C NMR spectrum of **7c**.

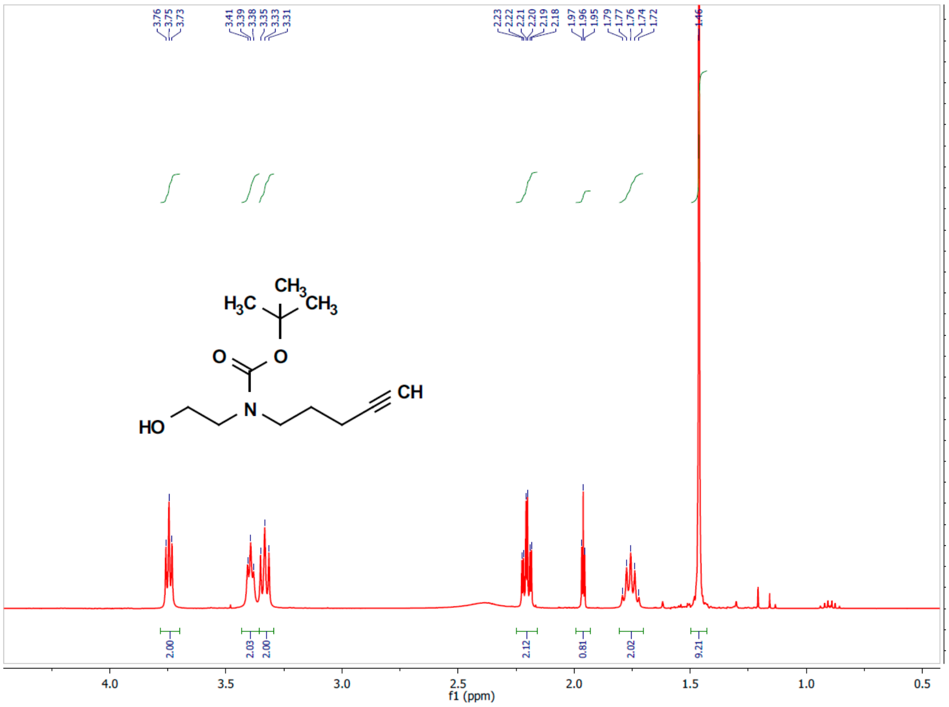

Figure S 25. ^{1}H NMR spectrum of **8a.**

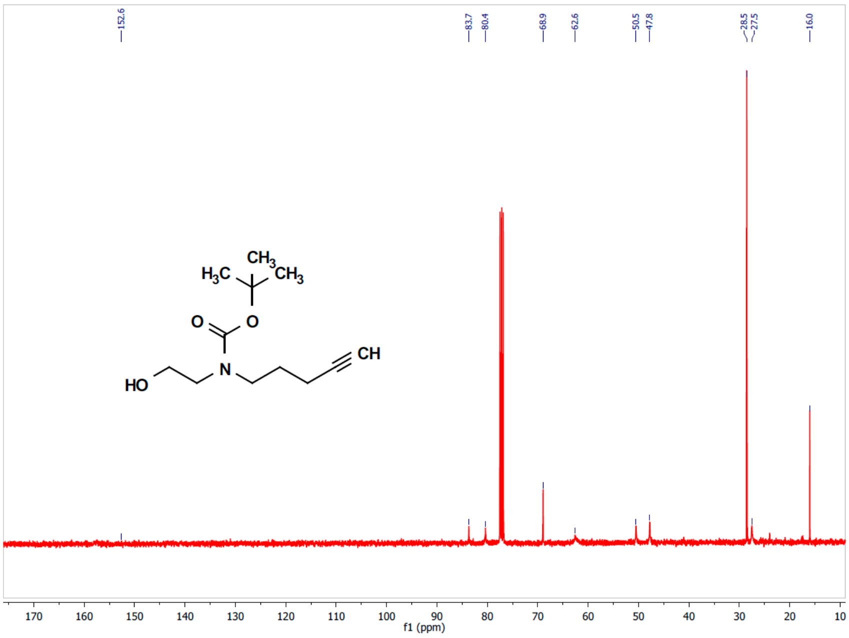

Figure S26. ^{13}C NMR spectrum of **8a**.

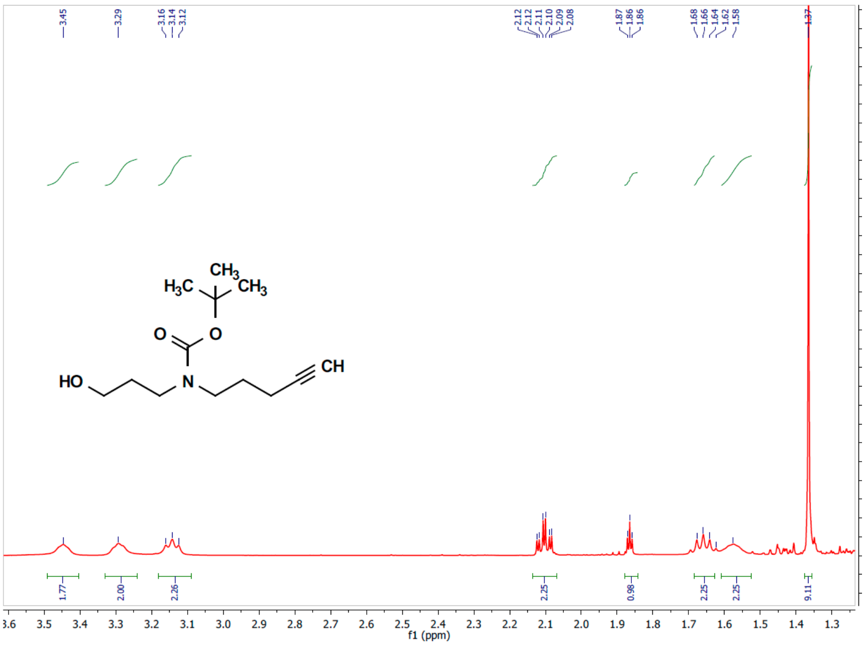

Figure S 27. ^{1}H NMR spectrum of **8b.**

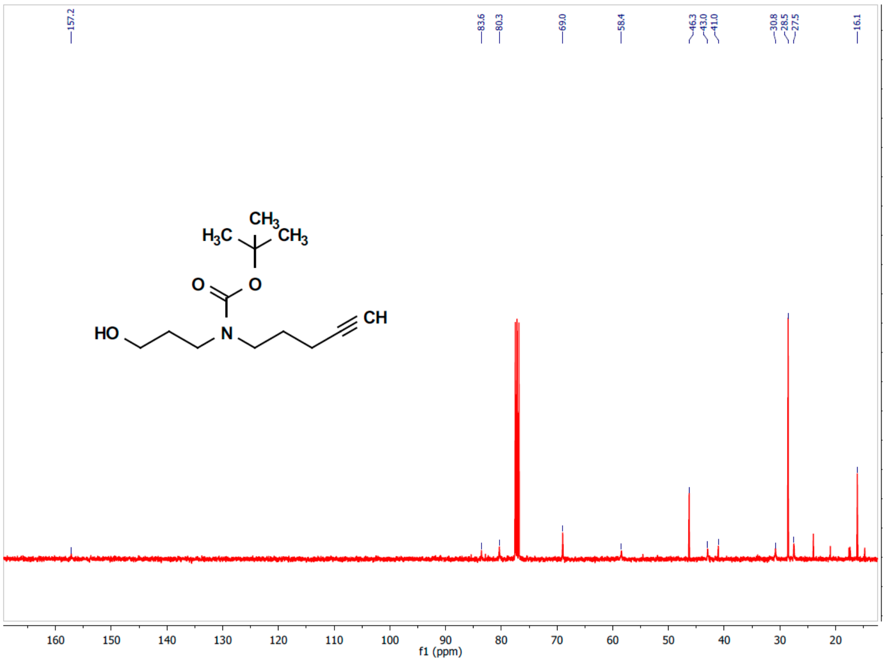

Figure S28. ^{13}C NMR spectrum of **8b**.

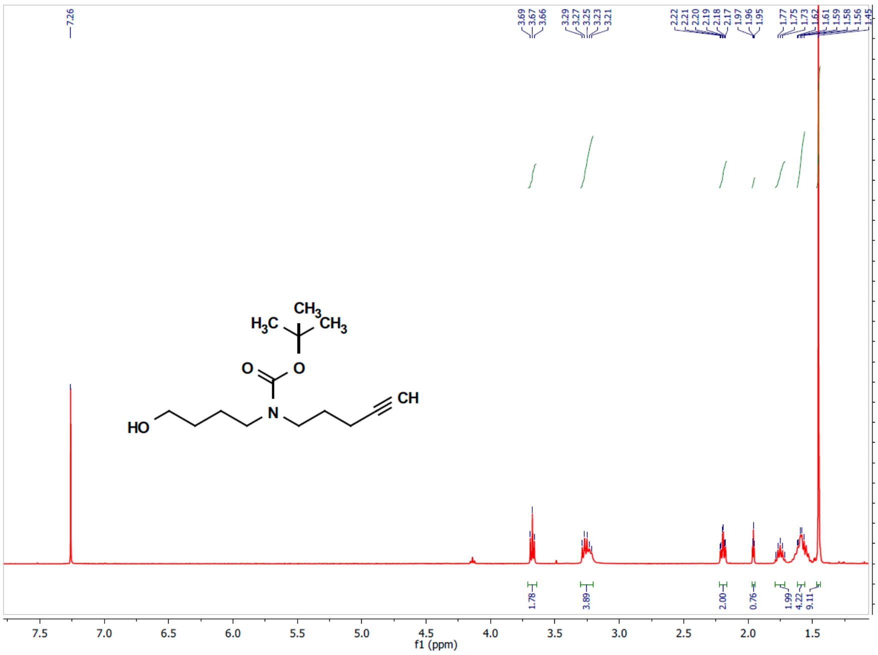

Figure S29. ^{1}H NMR spectrum of **8c.**

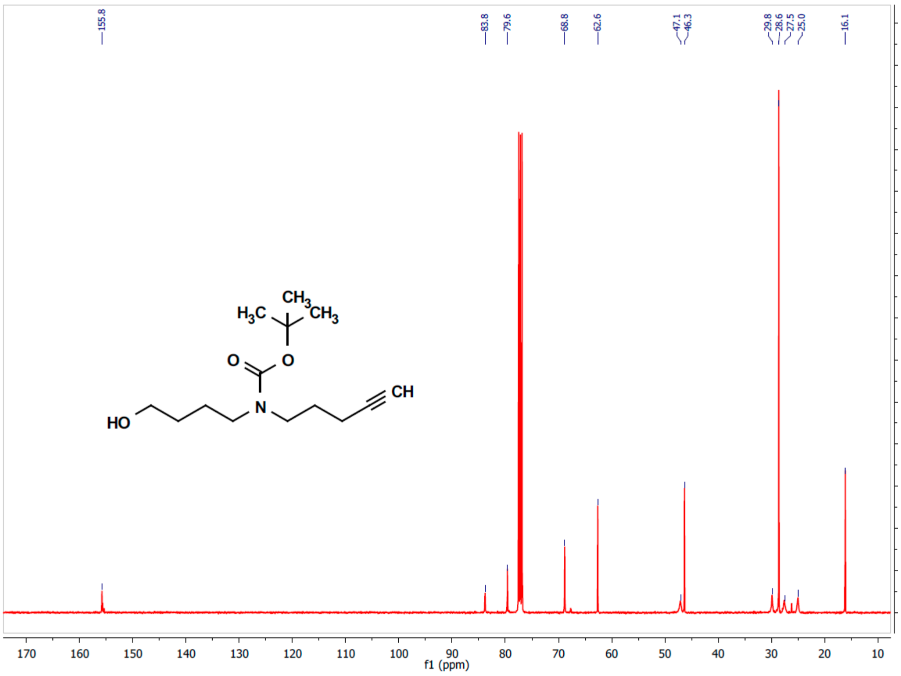

Figure S30. ^{13}C NMR spectrum of **8c**.

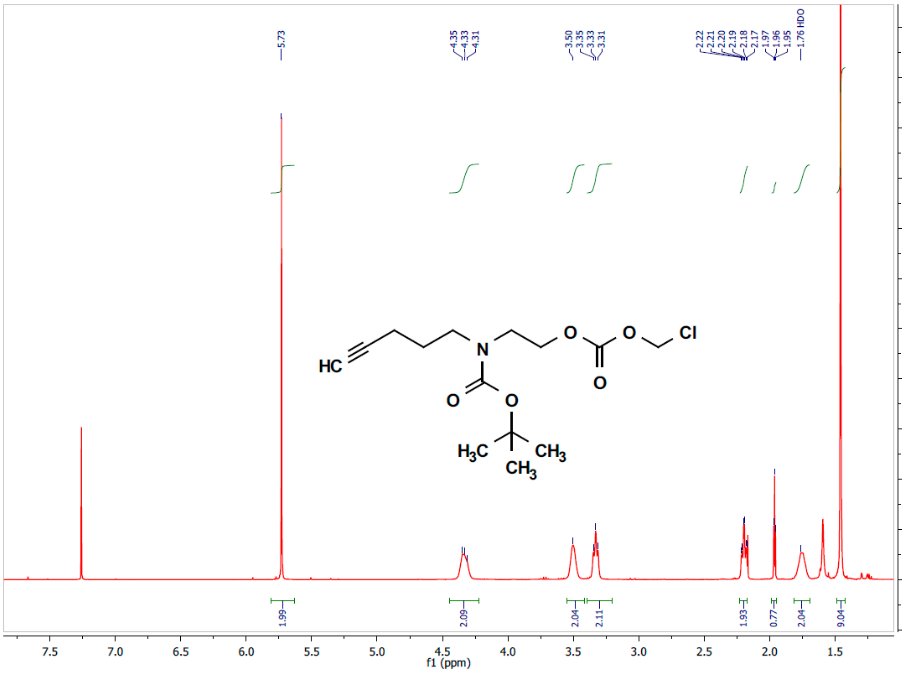

Figure S31. ^{1}H NMR spectrum of **9a**.

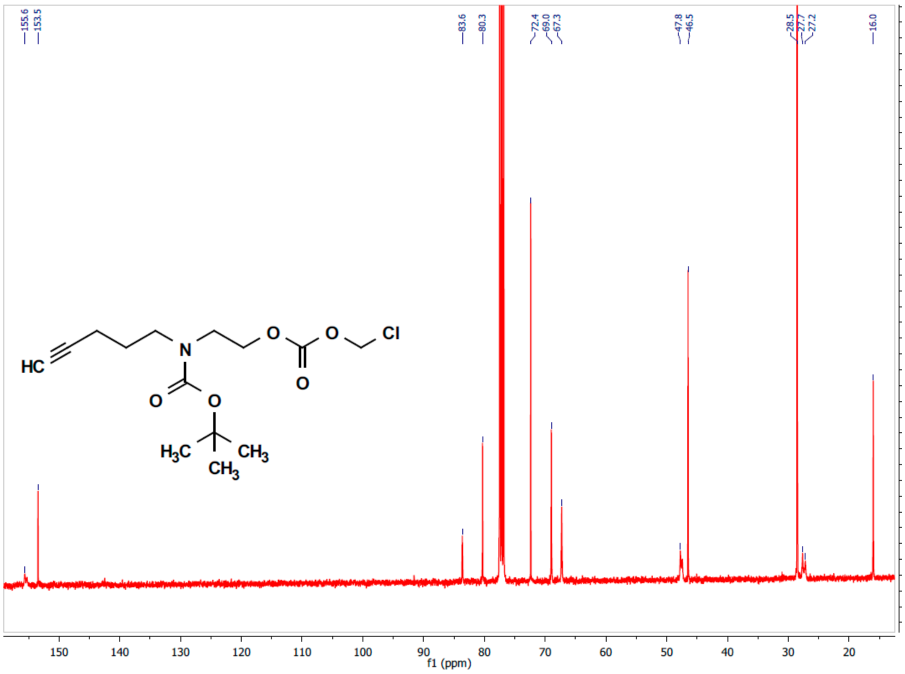

Figure S32. ^{13}C NMR spectrum of **9a**.

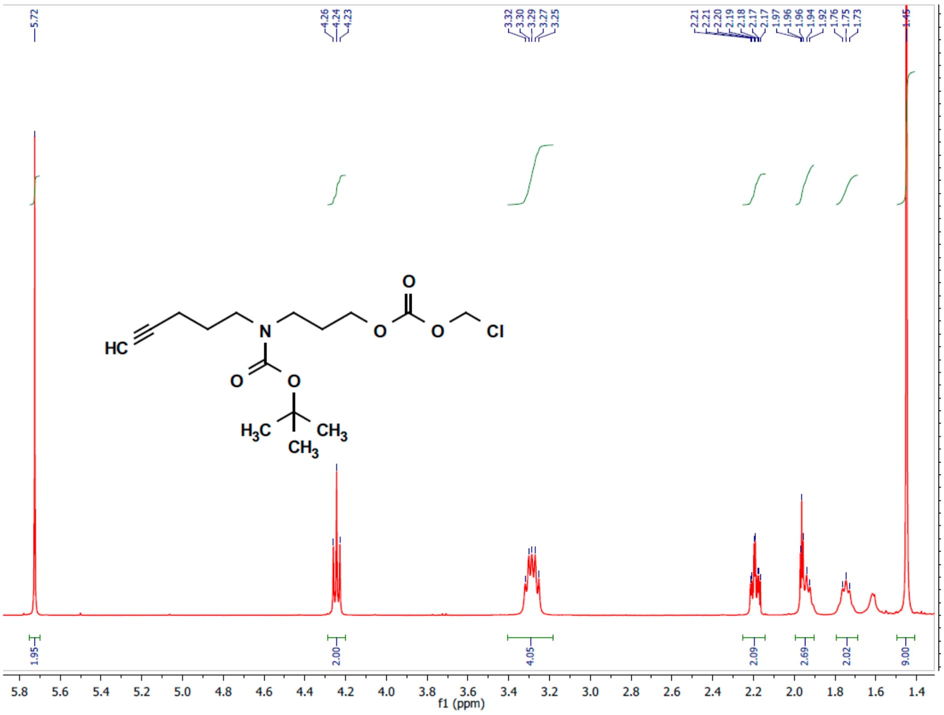

Figure S33. ^{1}H NMR spectrum of **9b** in $CDCl_3$.

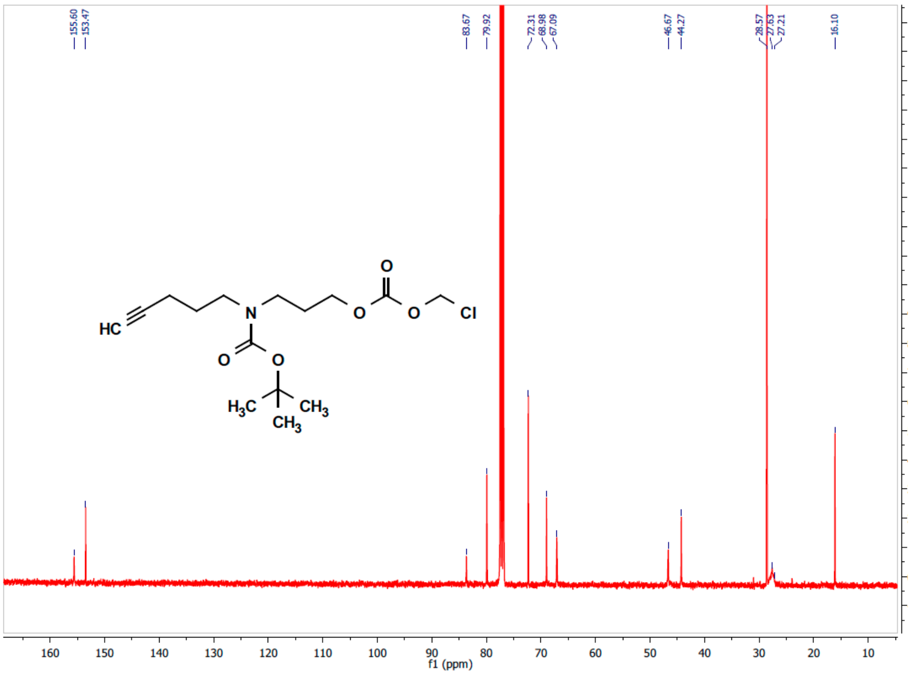

Figure S34. ^{13}C NMR spectrum of **9b** in $CDCl_3$**.**

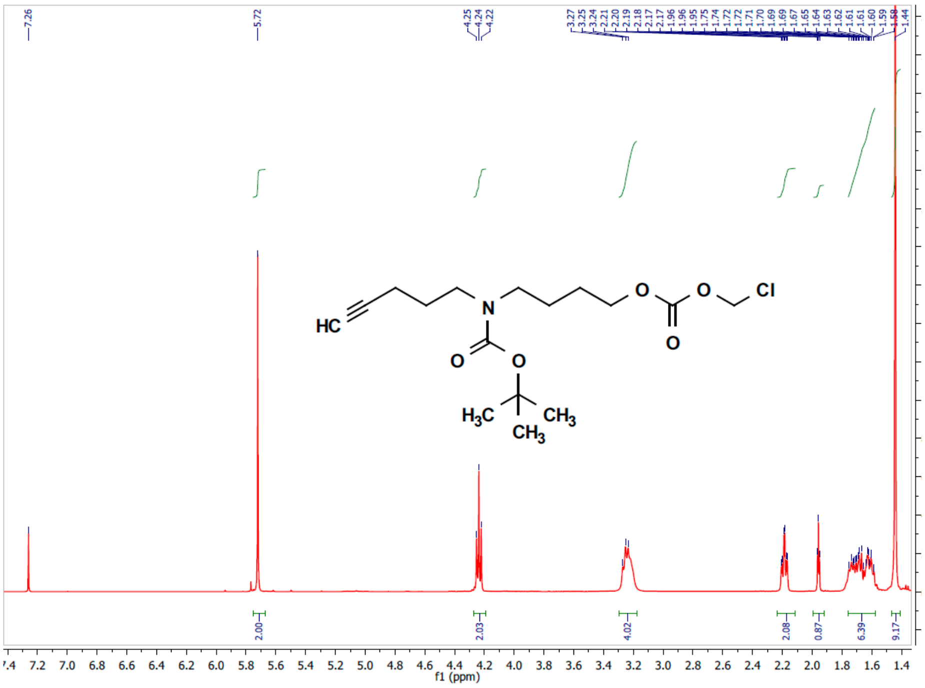

Figure S35. ^{1}H NMR spectrum of **9c** in $CDCl_3$.

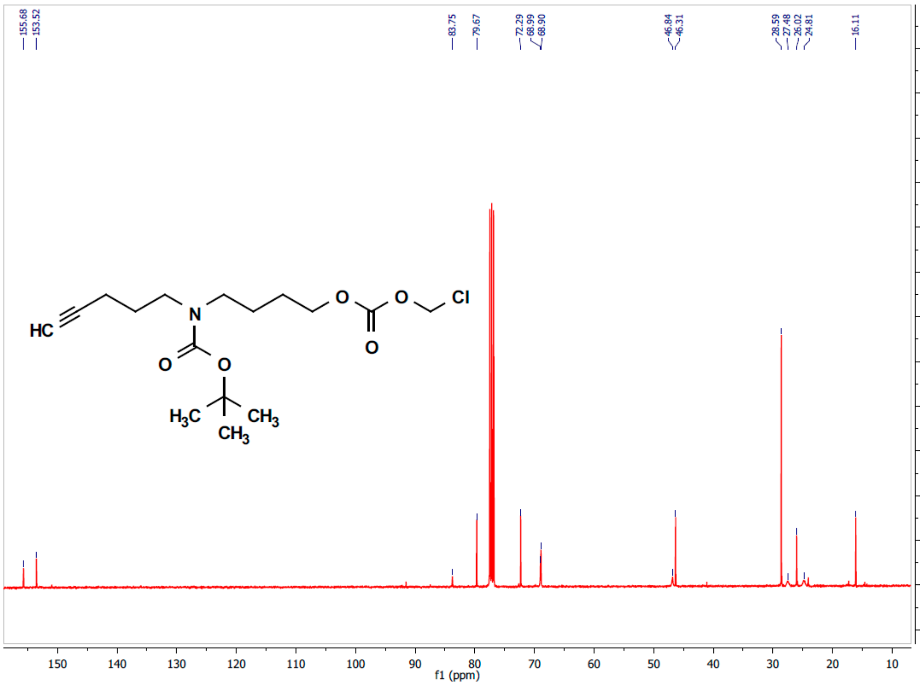

Figure S36. ^{13}C NMR spectrum of **9c** in $CDCl_3$**.**

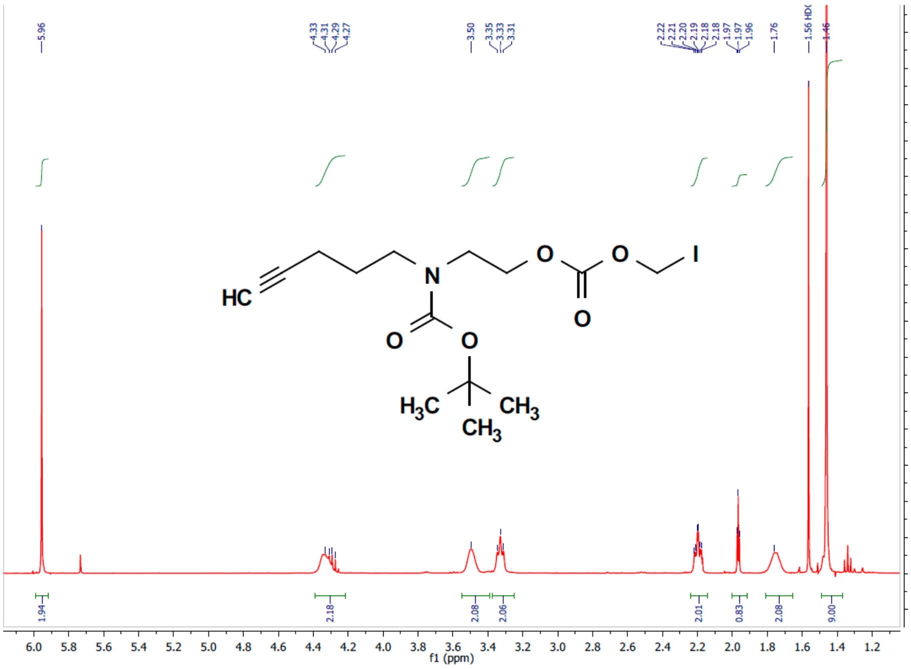

Figure S37. ^{1}H NMR spectrum of **10a** in $CDCl_3$.

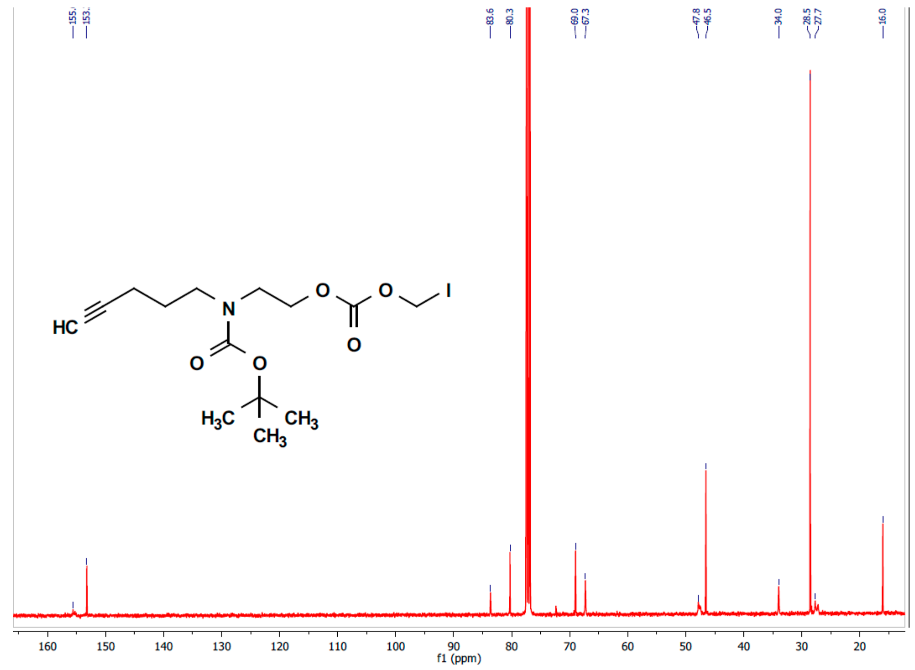

Figure S38. ^{13}C NMR spectrum of **10a** in $CDCl_3$**.**

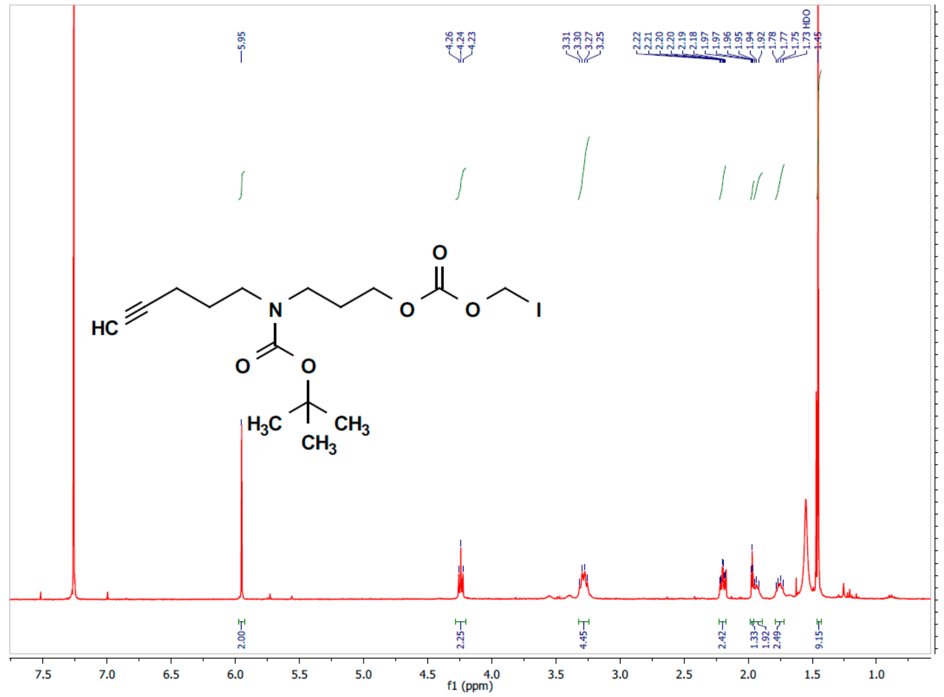

Figure S39. ^{1}H NMR spectrum of **10b** in $CDCl_3$.

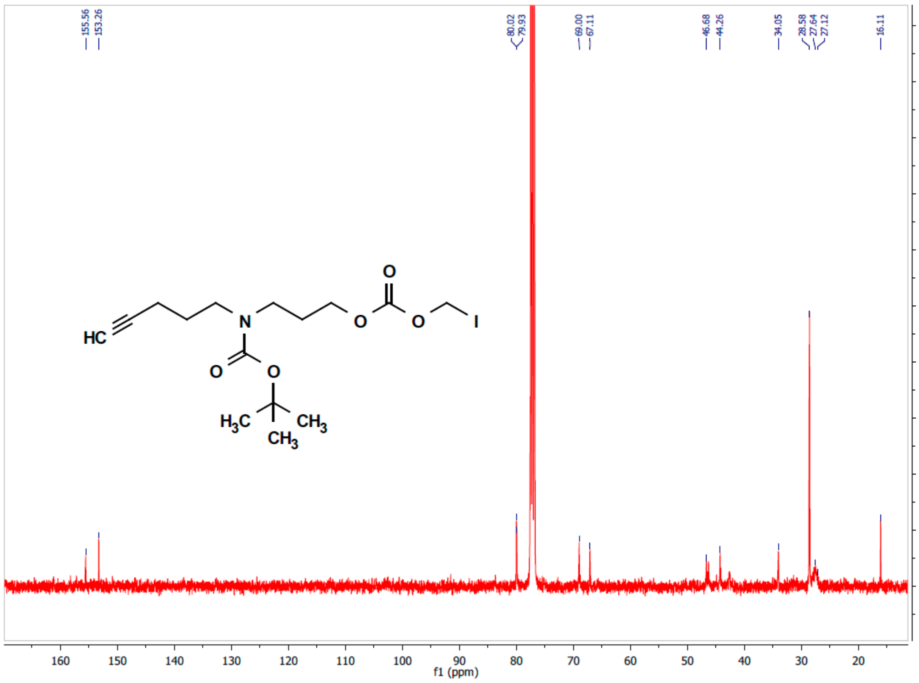

Figure S40. ^{13}C NMR spectrum of **10b** in CDCl3**.**

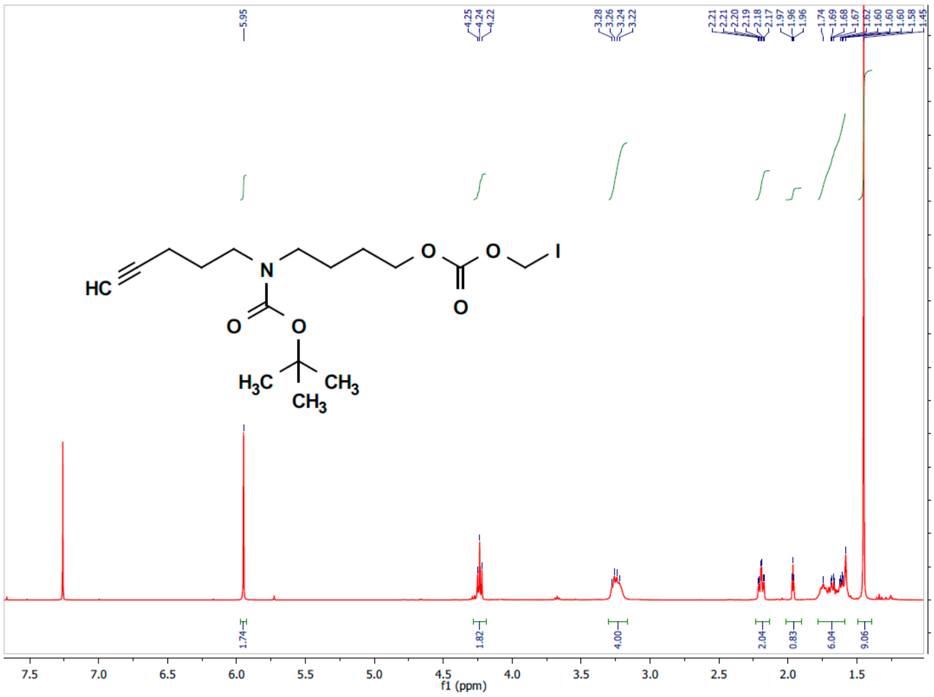

Figure S41. ^{1}H NMR spectrum of **10c** in CDCl3.

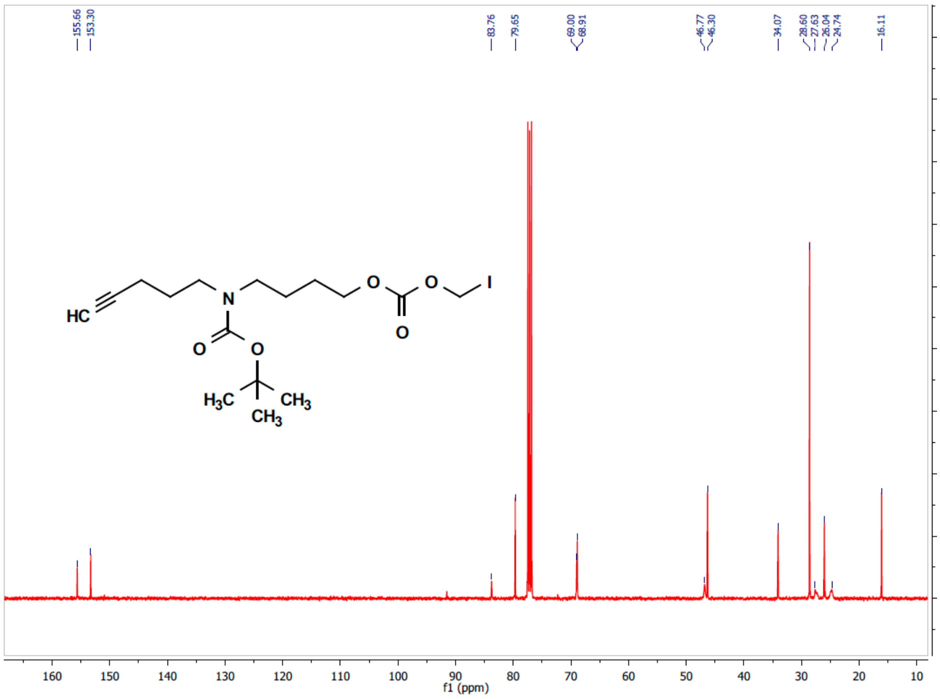

Figure S42. ^{13}C NMR spectrum of **10c** in $CDCl_3$**.**

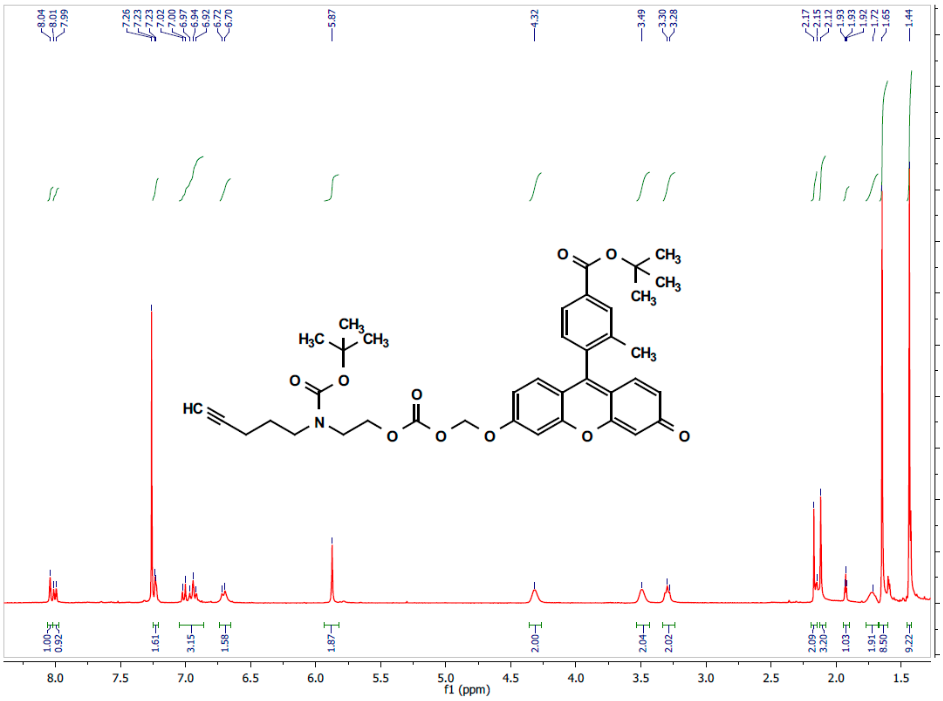

Figure S43. ^{1}H NMR spectrum of **11a** in $CDCl_3$.

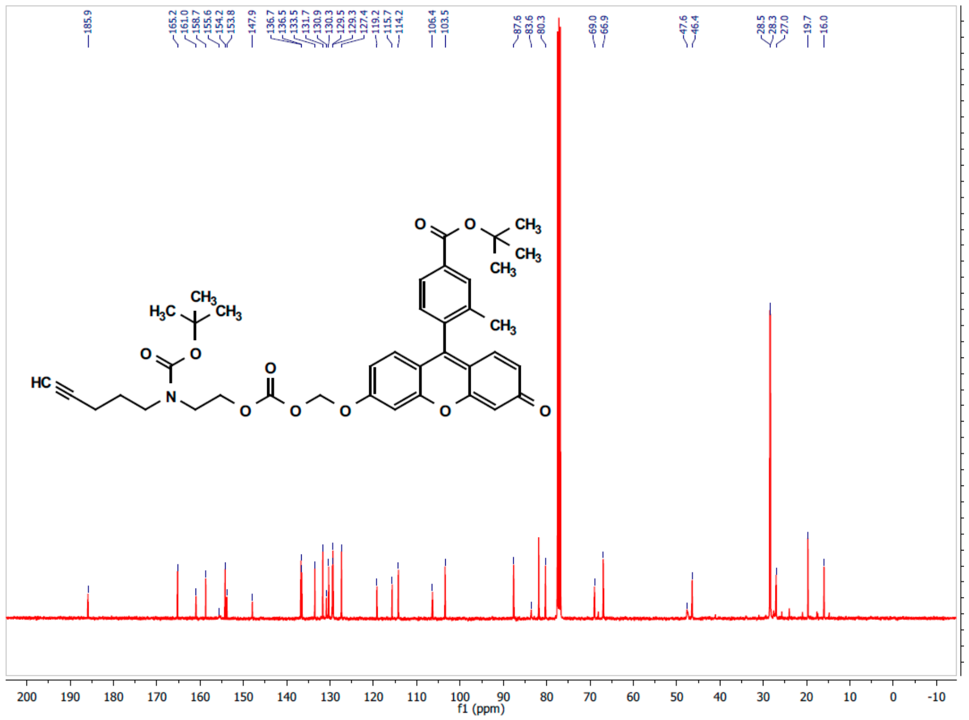

Figure S44. ^{13}C NMR spectrum of **11a** in $CDCl_3$**.**

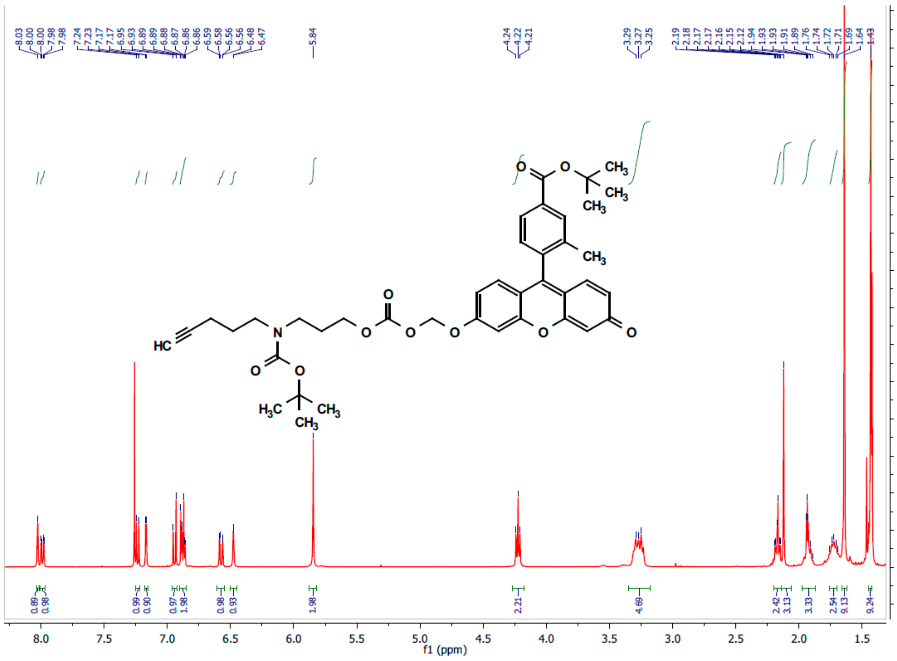

Figure S45. ^{1}H NMR spectrum of **11b** in $CDCl_3$.

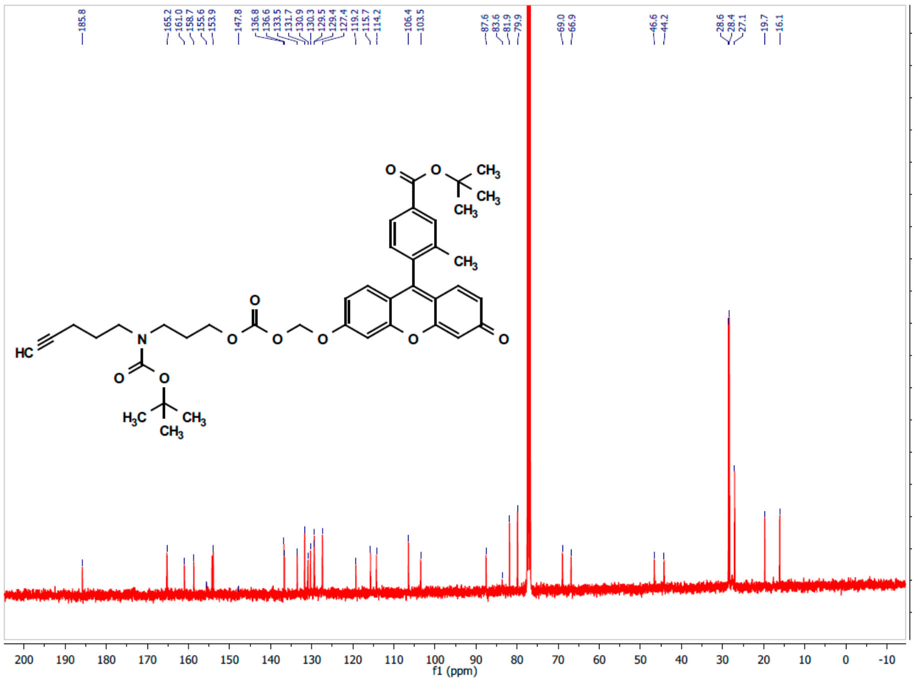

Figure S46. ^{13}C NMR spectrum of **11b** in $CDCl_3$**.**

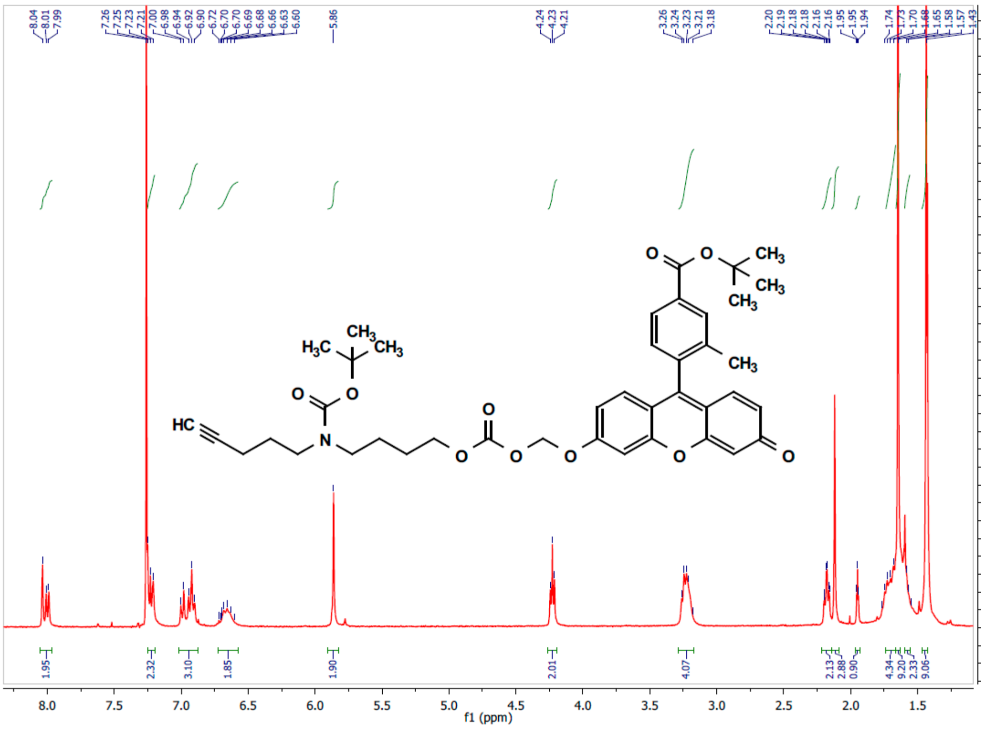

Figure S47. ^{1}H NMR spectrum of **11c** in $CDCl_3$.

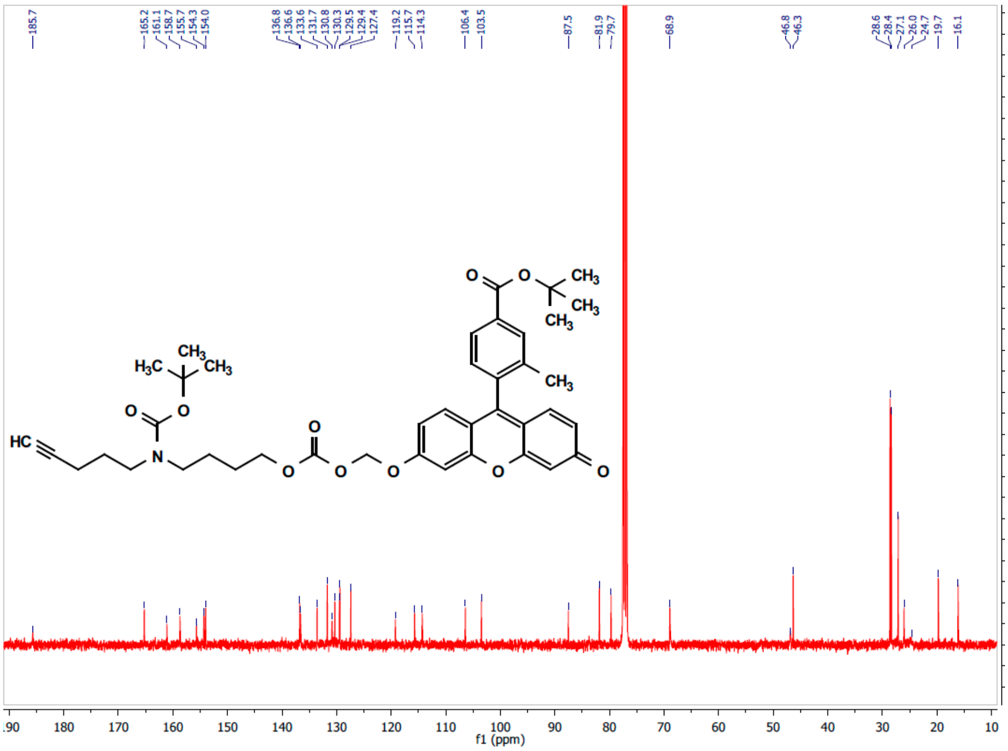

Figure S48. ^{13}C NMR spectrum of **11c** in $CDCl_3$.

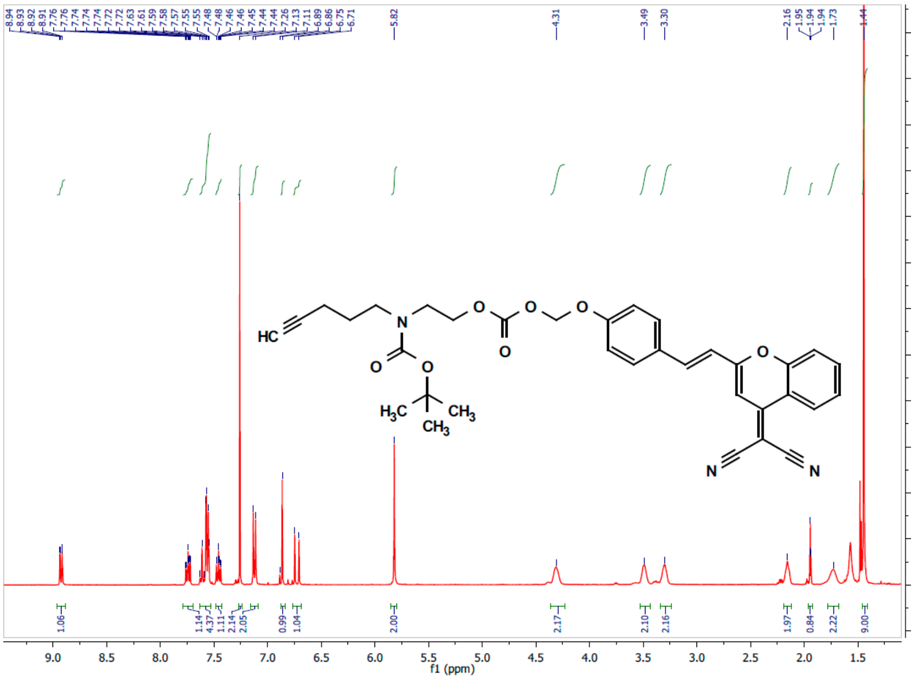

Figure S49. ^{1}H NMR spectrum of **12a** in $CDCl_3$.

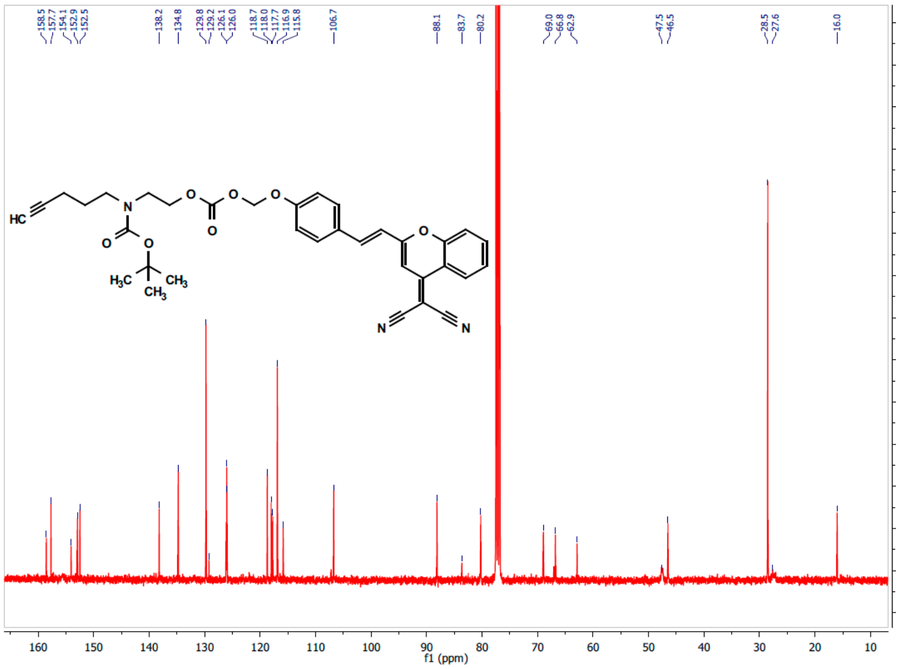

Figure S50. ^{13}C NMR spectrum of **12a** in $CDCl_3$**.**

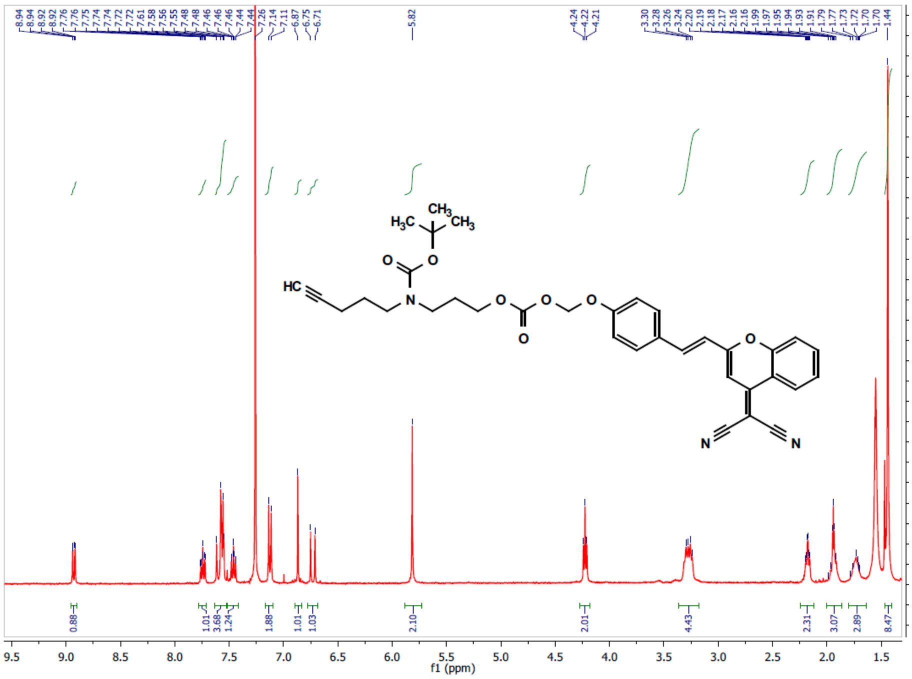

Figure S51. ^{1}H NMR spectrum of **12b** in $CDCl_3$

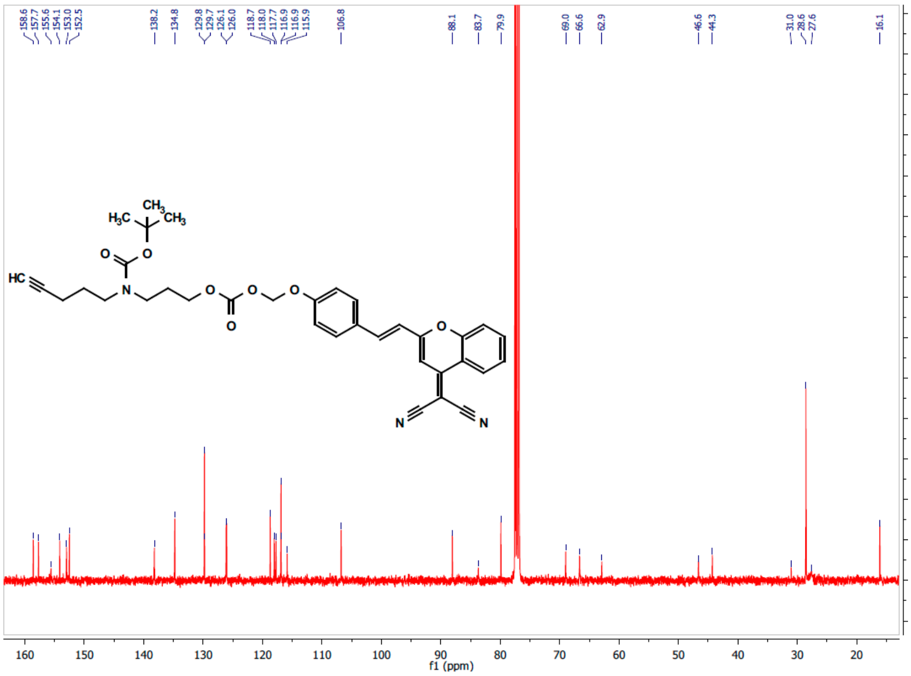

Figure S52. ^{13}C NMR spectrum of **12b** in $CDCl_3$.

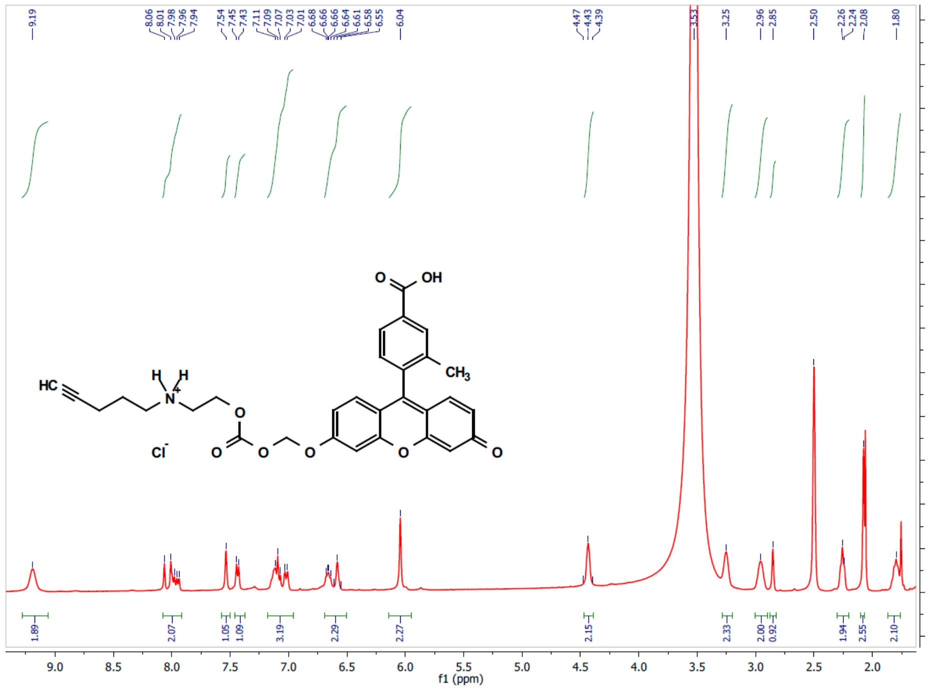

Figure S53. ^{1}H NMR spectrum of Probe **13** in DMSO-d$_6$.

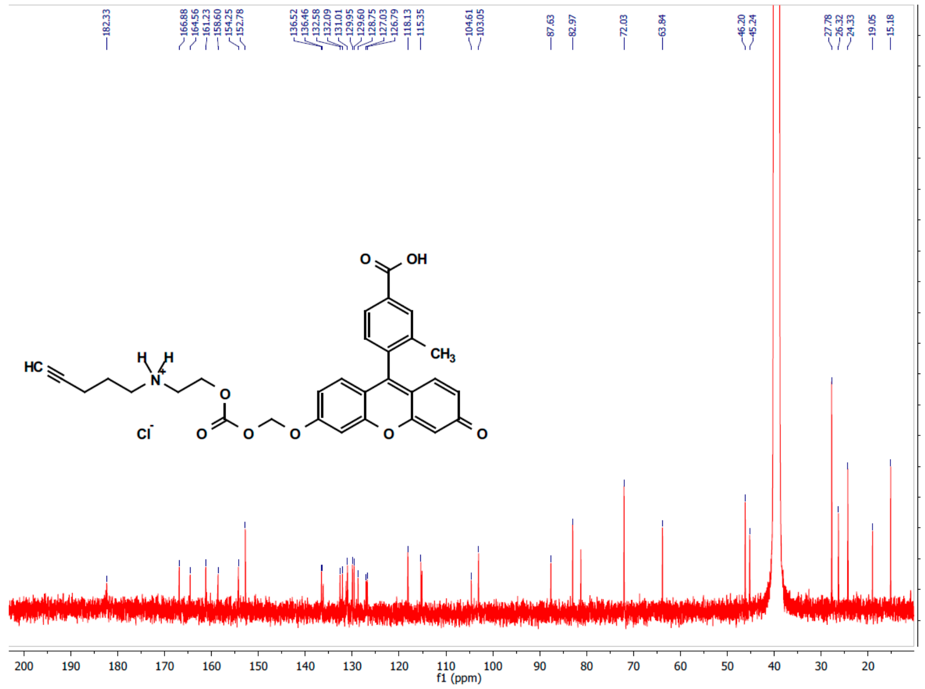

Figure S54. ^{13}C NMR spectrum of Probe **13** in DMSO-d$_6$.

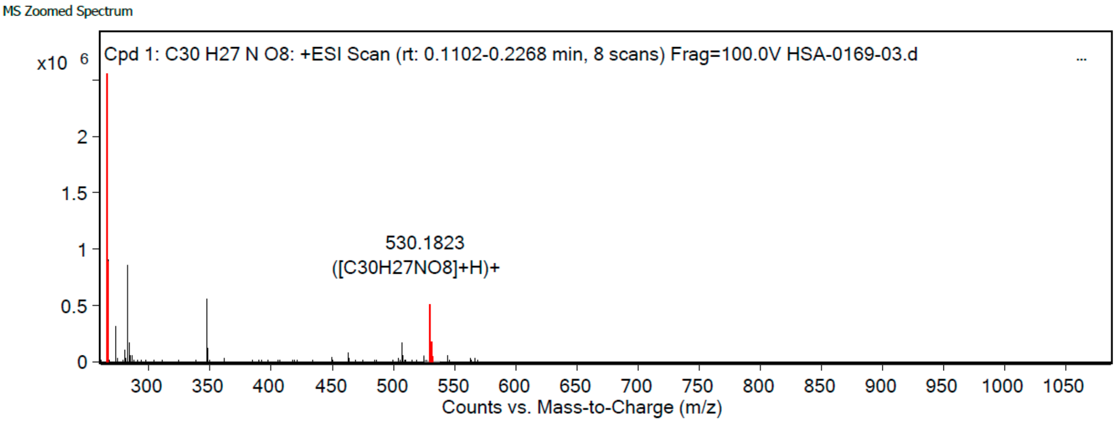

Figure S55. HRMS of probe **13.**

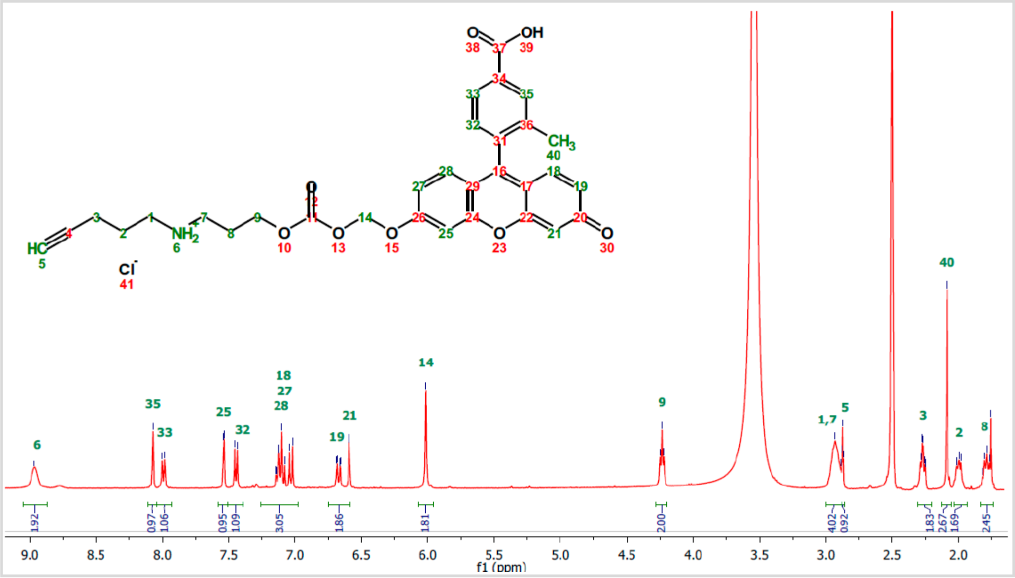

Figure S56. ^{1}H NMR spectrum of Probe **14** in DMSO-d_6.

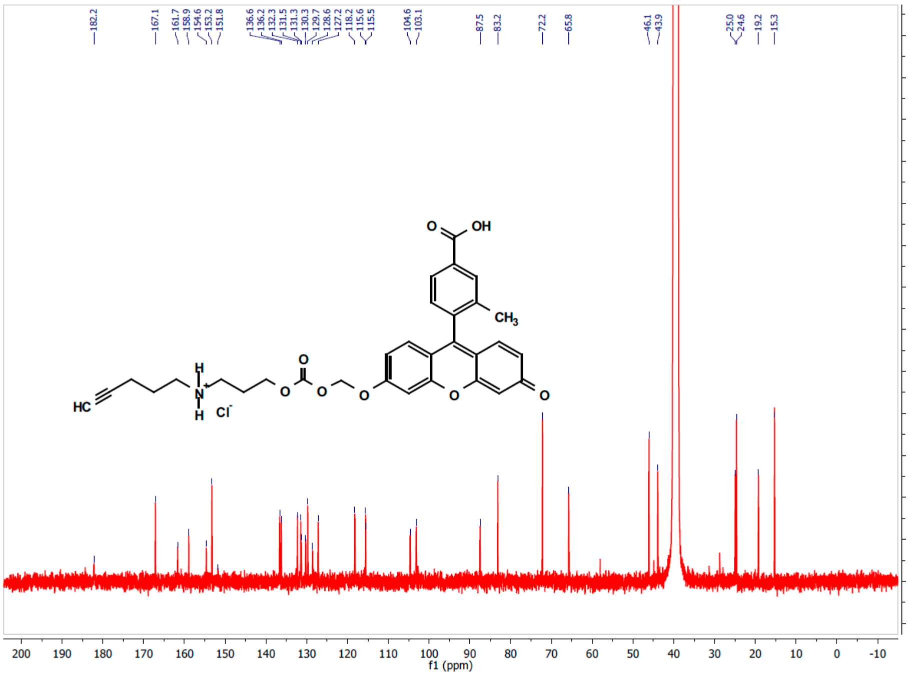

Figure S57. ^{13}C NMR spectrum of Probe **14** in DMSO-d$_6$.

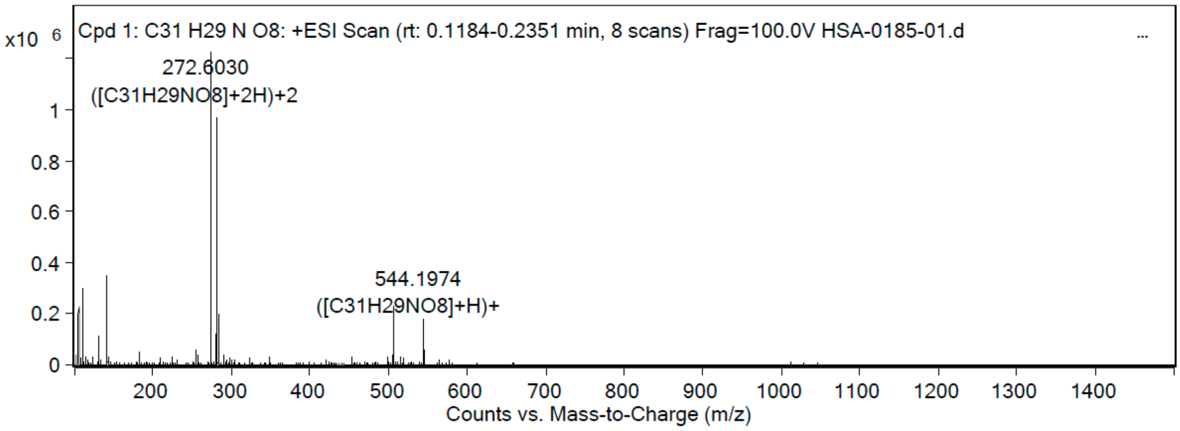

Figure S58. HRMS of probe **14.**

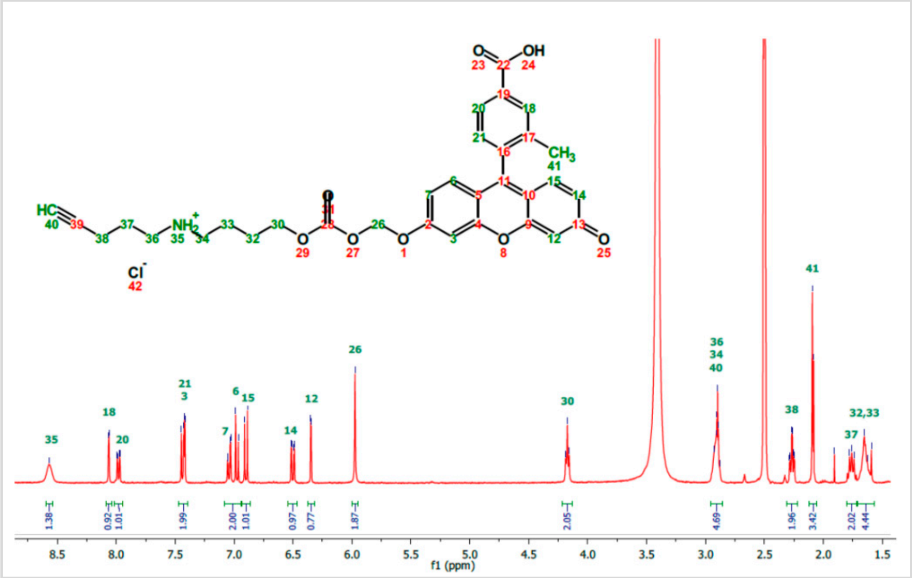

Figure S59. ^{1}H NMR spectrum of probe **15** in DMSO-d$_6$.

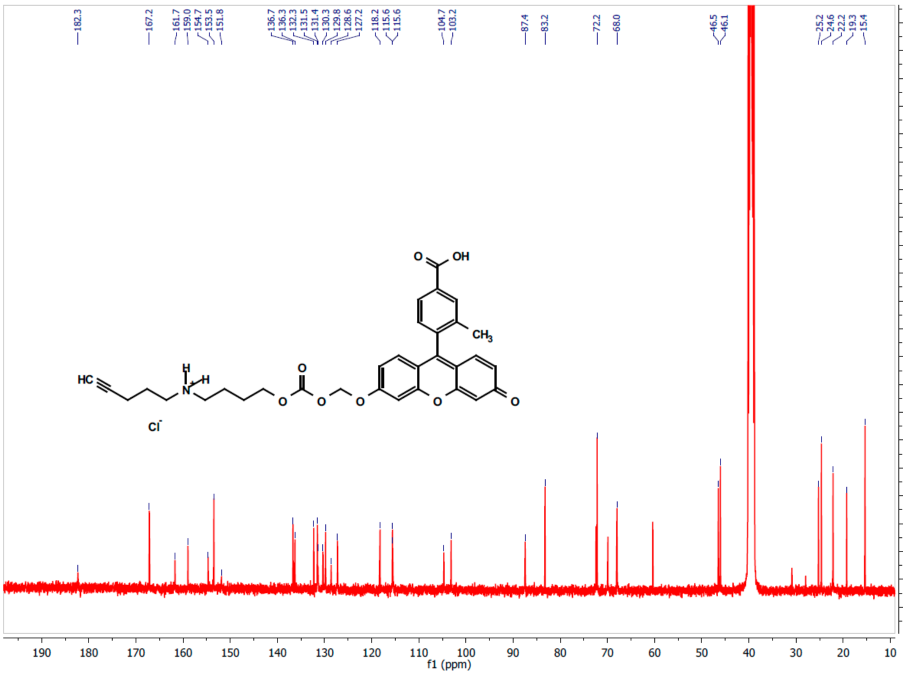

Figure S60. ^{13}C NMR spectrum of Probe **15** in DMSO-d$_6$.

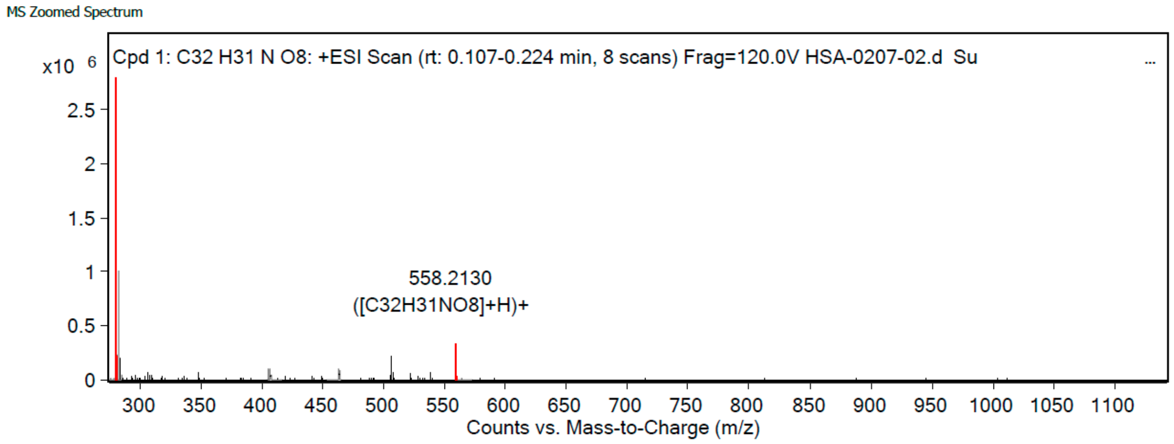

Figure S61. HRMS of Probe **15**.

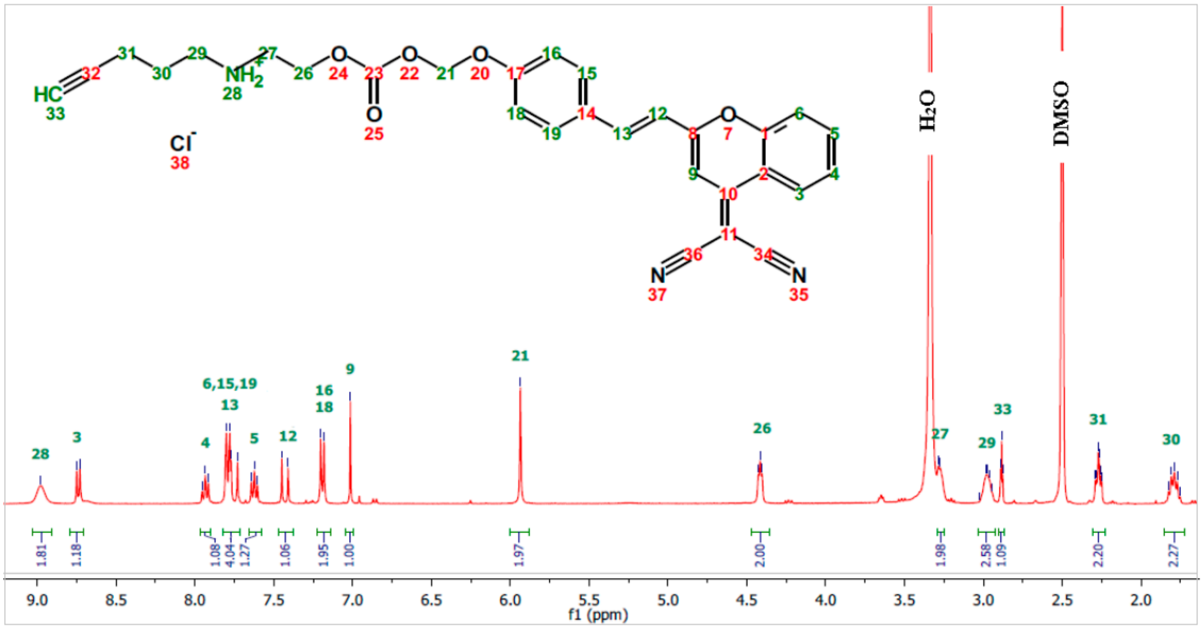

Figure S62. ^{1}H NMR spectrum of Probe **16** in DMSO-d$_6$**.**

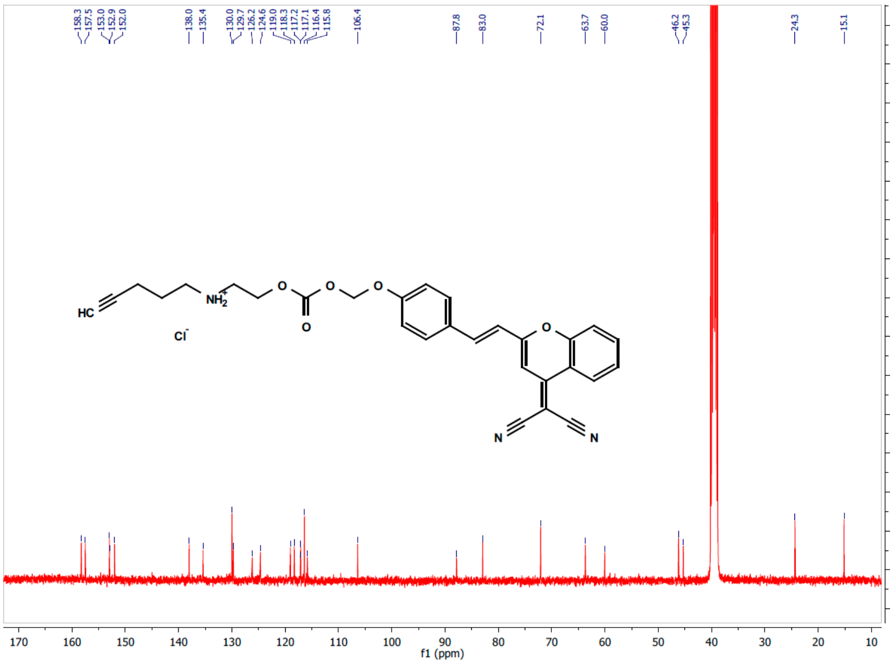

Figure S63.^{13}C NMR spectrum of probe **16** in DMSO-d_6**.**

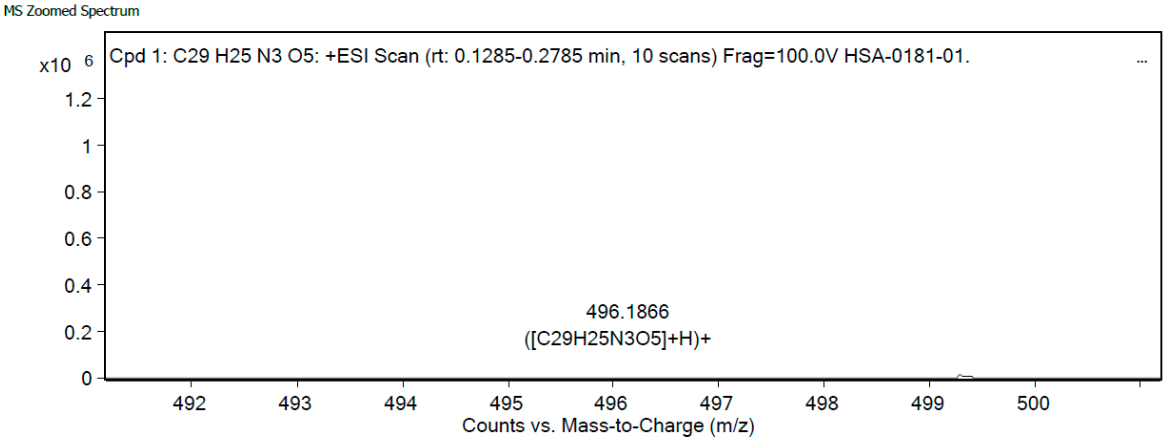

Figure S64. HRMS of Probe **16**.

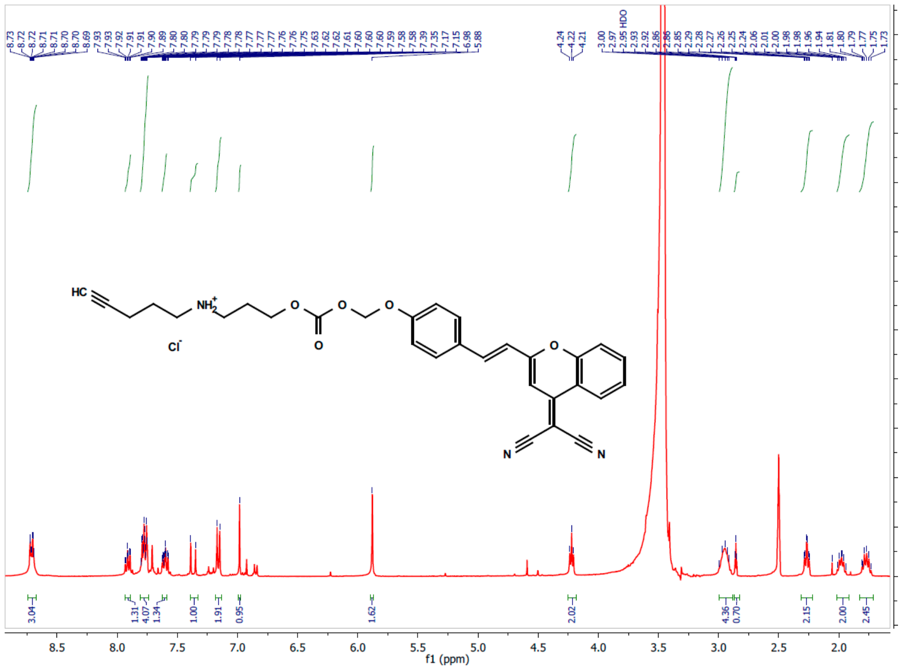

Figure S65. ^{1}H NMR spectrum of Probe **17** in DMSO-d_6.

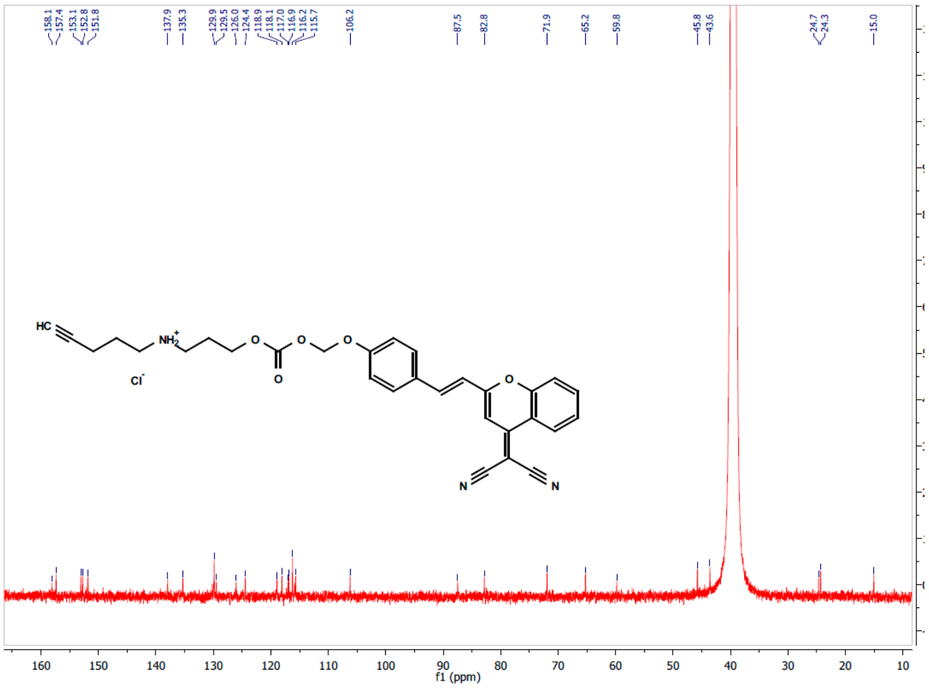

Figure S66. ^{13}C NMR spectrum of probe **17** in DMSO-d_6.

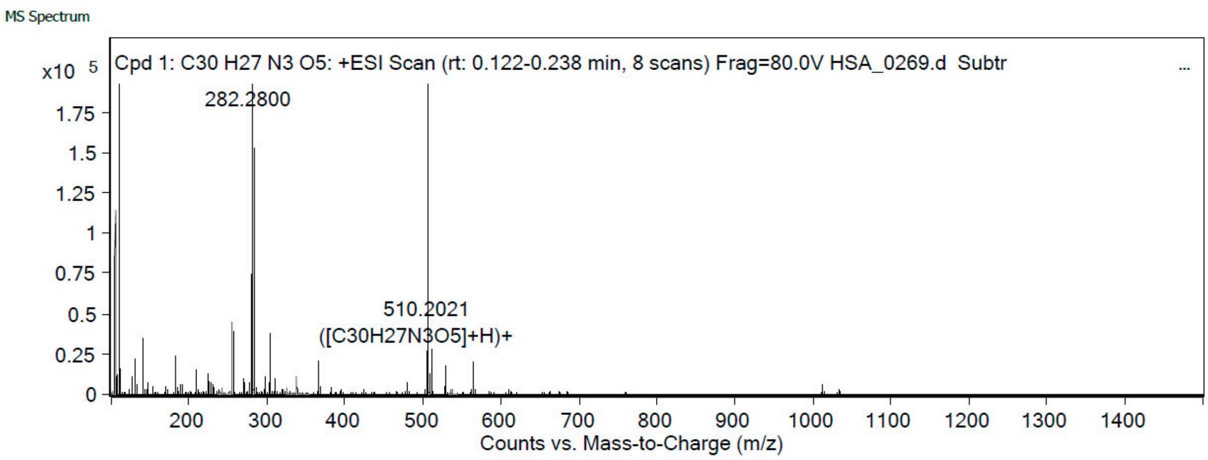

Figure S67. HRMS of Probe **17**.

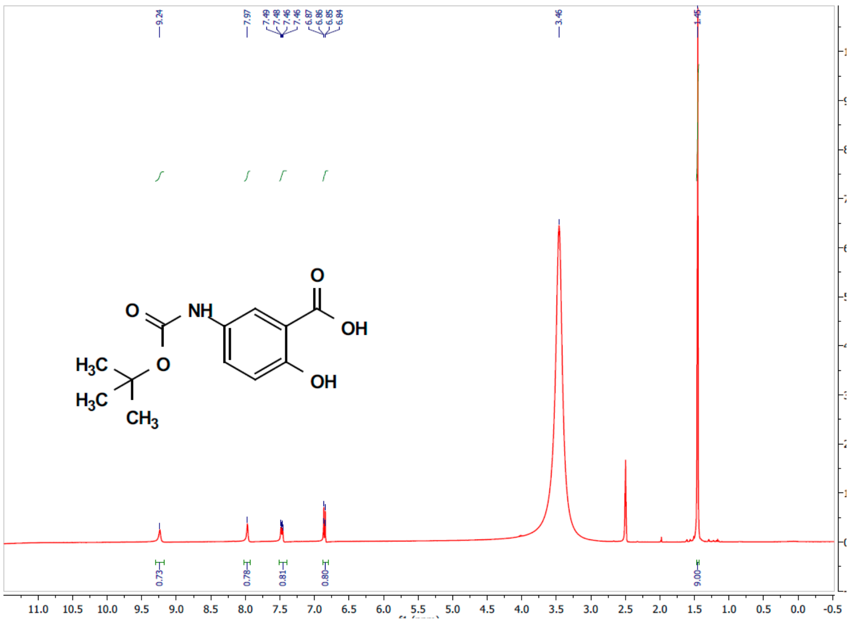

Figure S68. ^{1}H NMR spectrum of **18** in DMSO-d6.

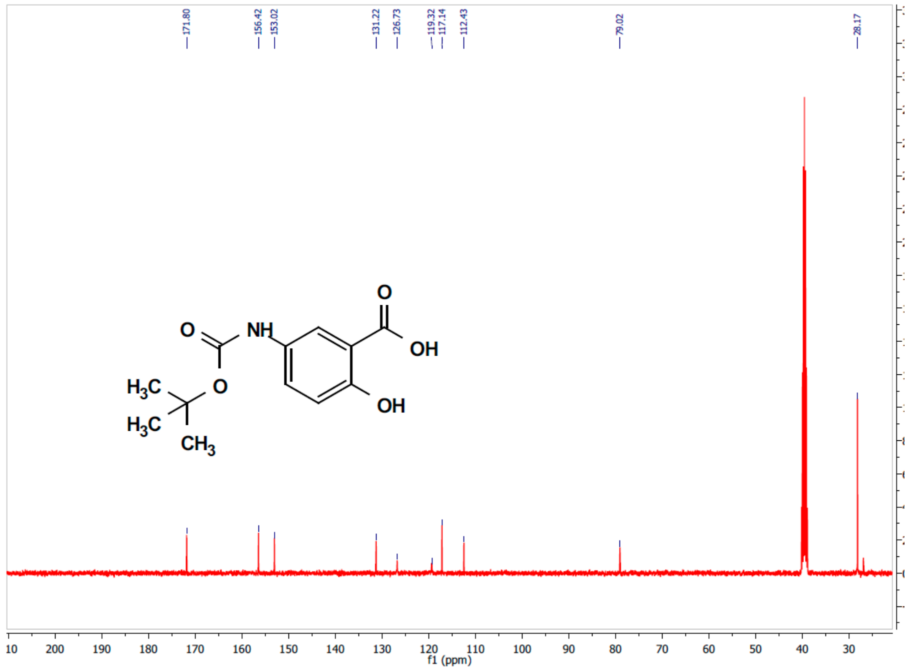

Figure S69. ^{13}C NMR spectrum of **18** in DMSO-d_6.

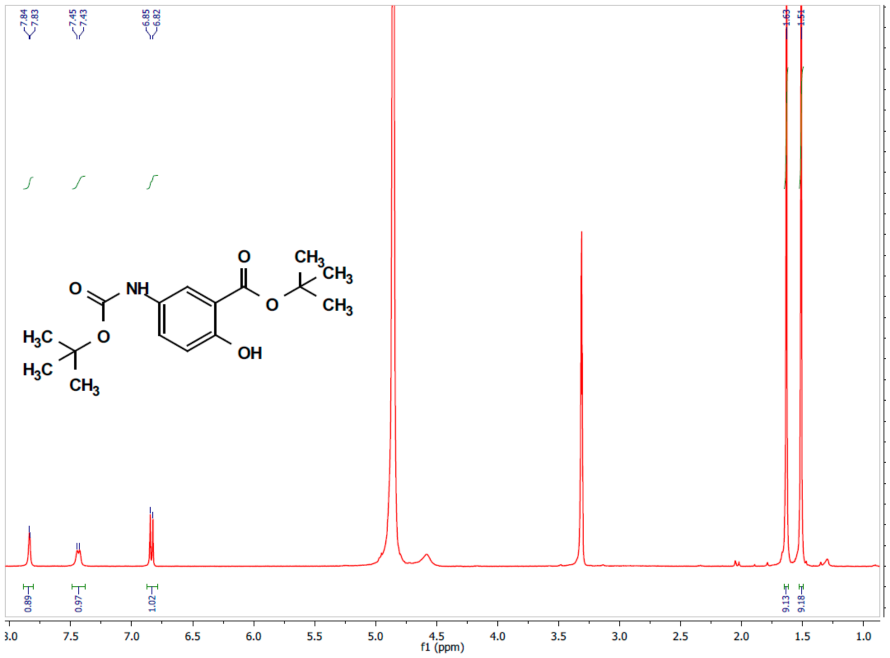

Figure S70. ^{1}H NMR spectrum of **19** in MeOD.

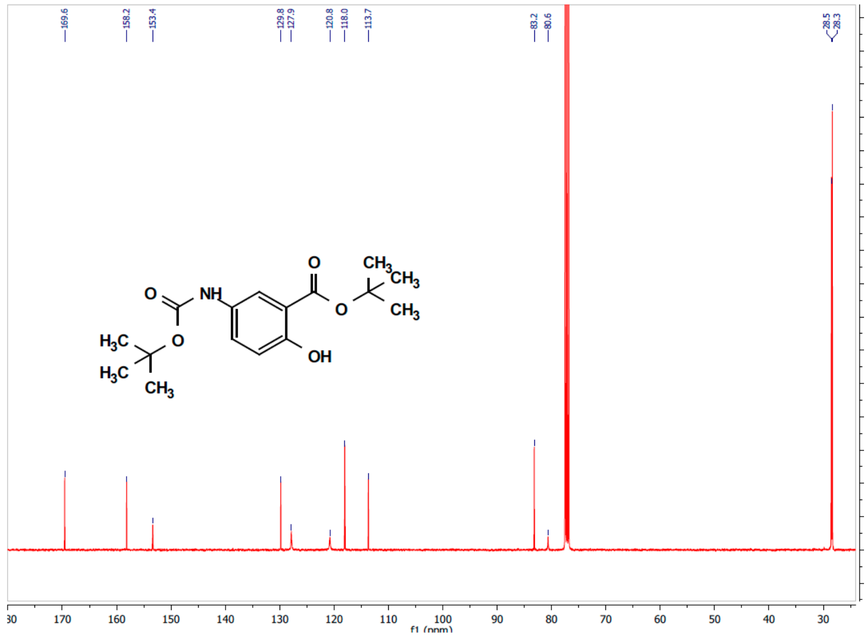

Figure S71. ^{13}C NMR spectrum of **19** in $CDCl_3$.

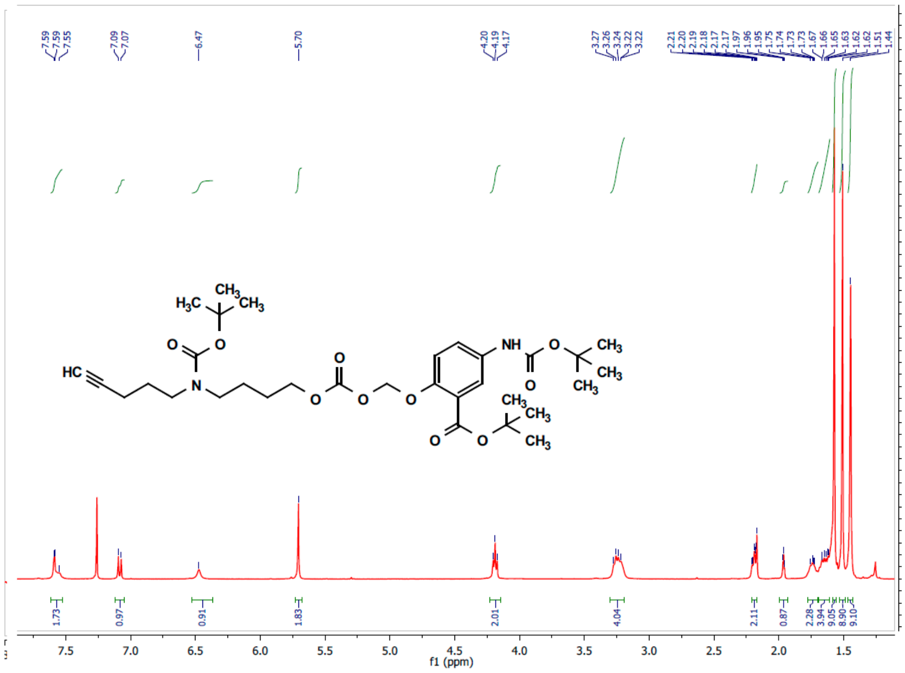

Figure S72. ^{1}H NMR spectrum of **20a** in $CDCl_3$.

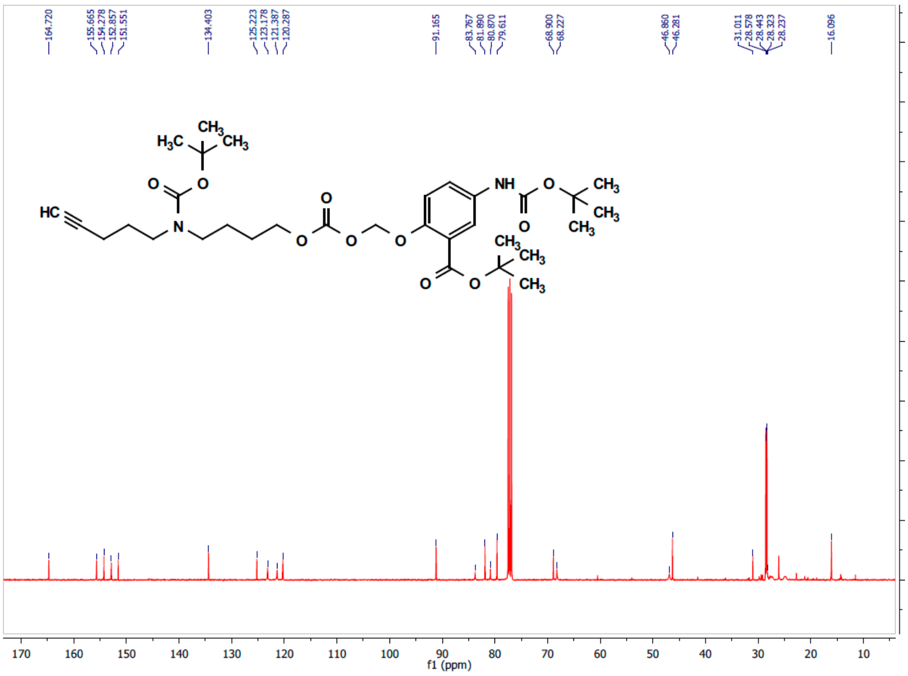

Figure S73. ^{13}C NMR spectrum of **20a** in $CDCl_3$**.**

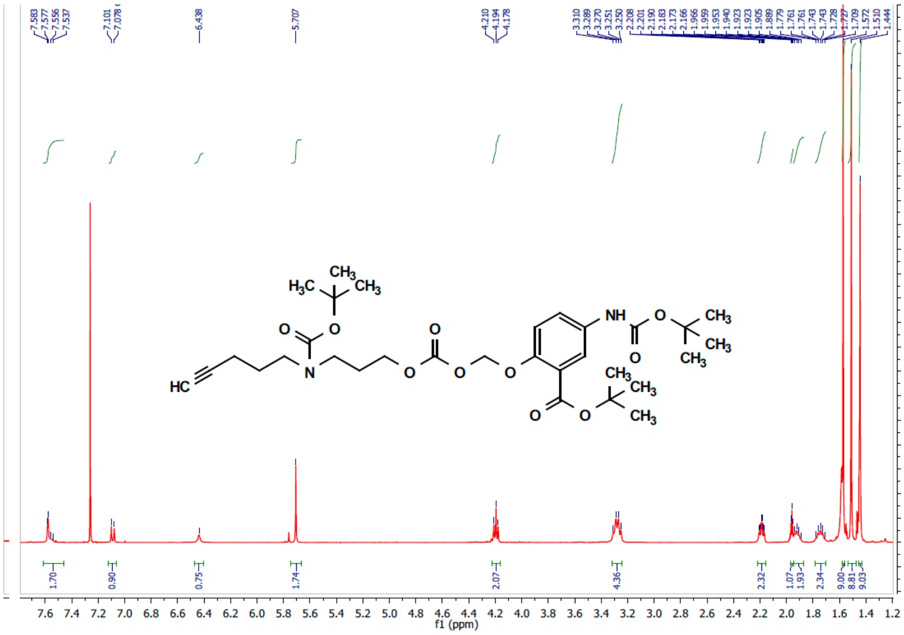

Figure S74. ^{1}H NMR spectrum of **20b** in $CDCl_3$.

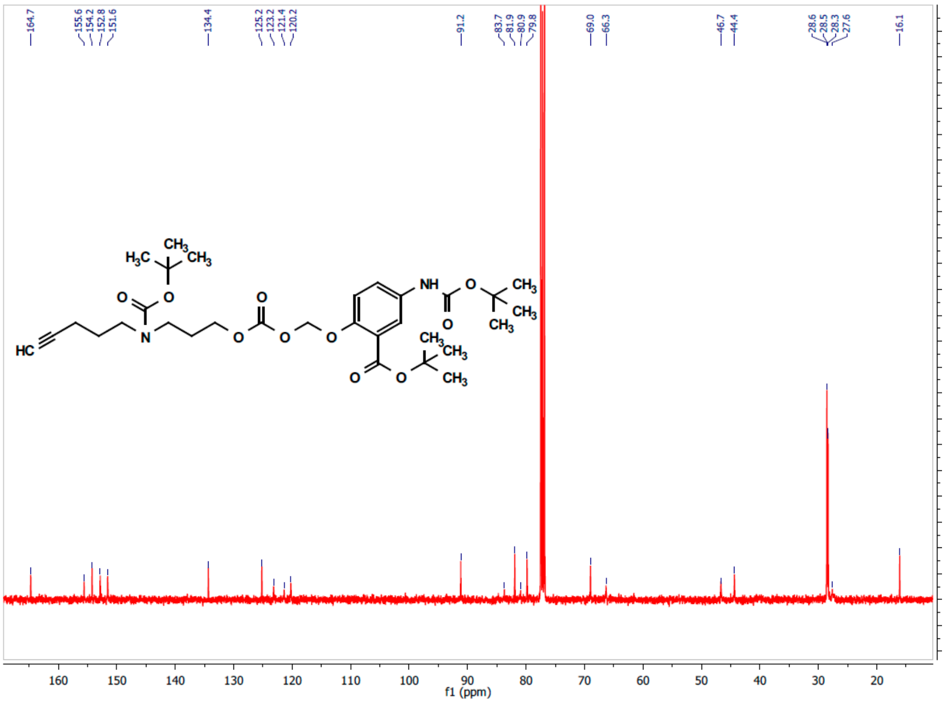

Figure S75. ^{13}C NMR spectrum of **20b** in $CDCl_3$**.**

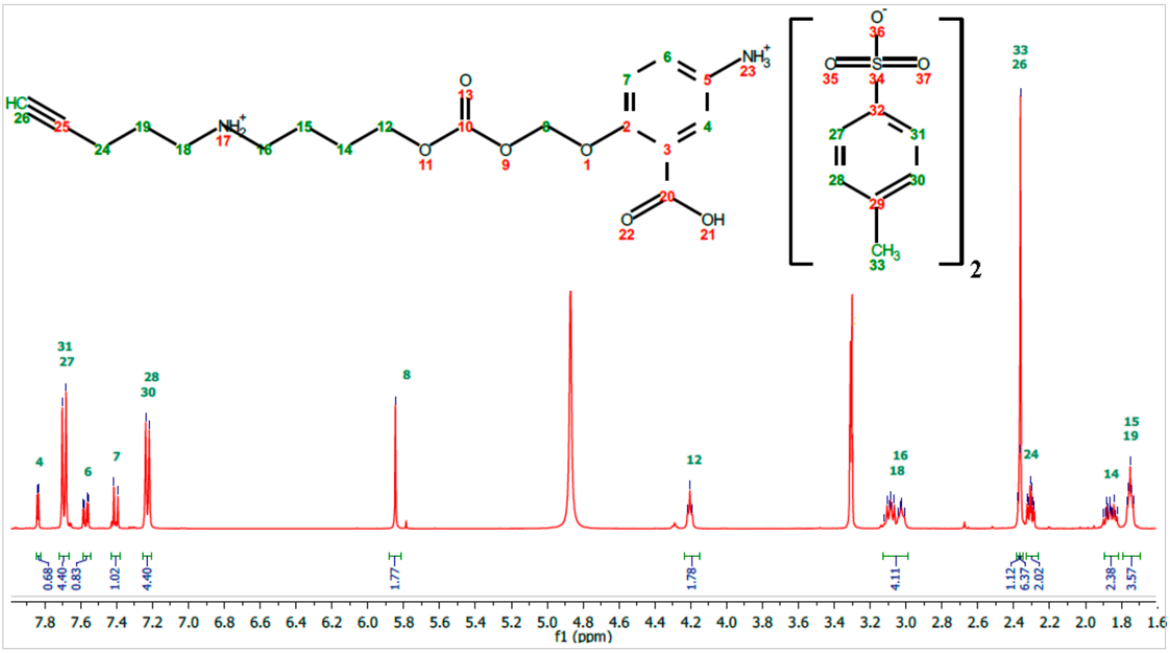

Figure S76. ^{1}H NMR spectrum of Msl prodrug **21** in MeOD.

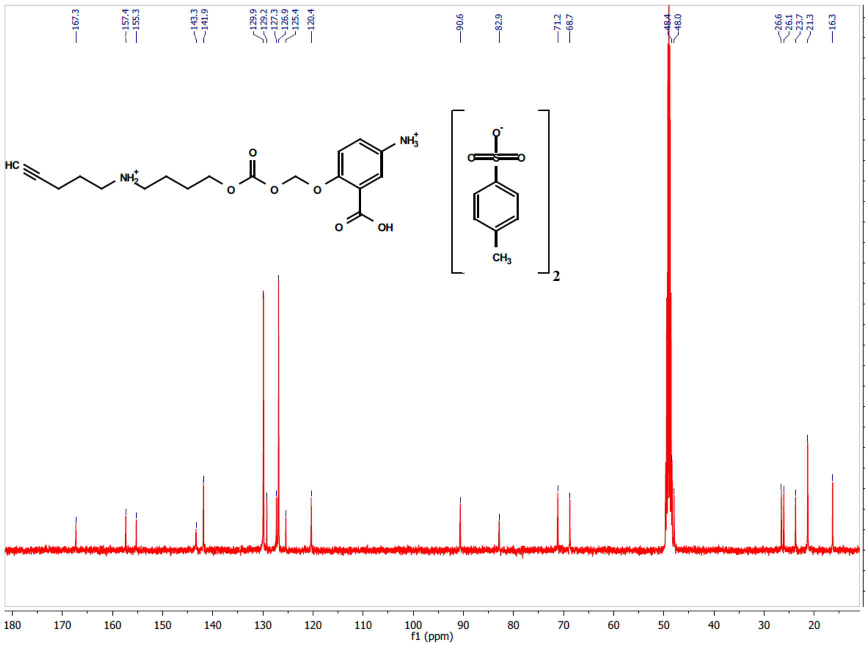

Figure S77. ^{13}C NMR spectrum of Msl prodrug **21** in MeOD.

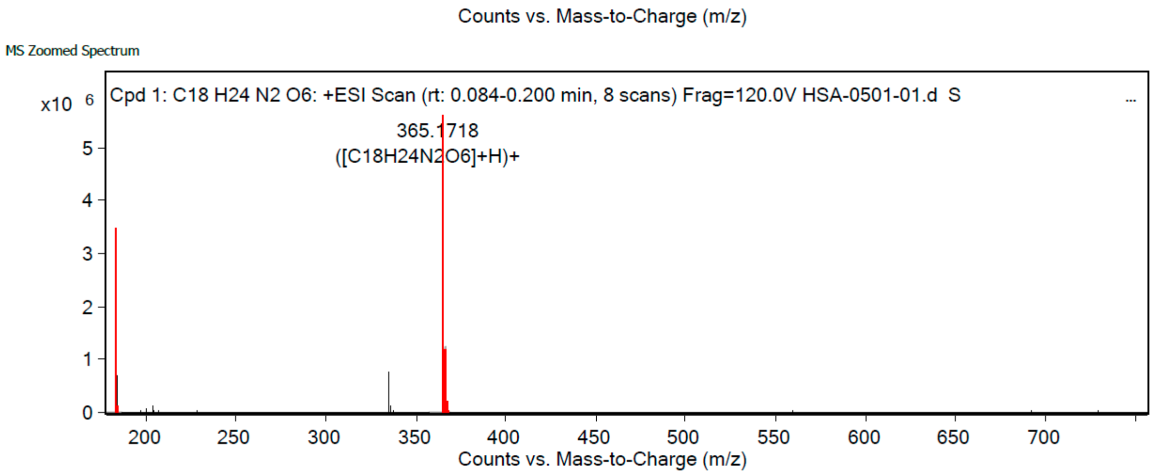

Figure S78. HRMS of Msl prodrug **21.**

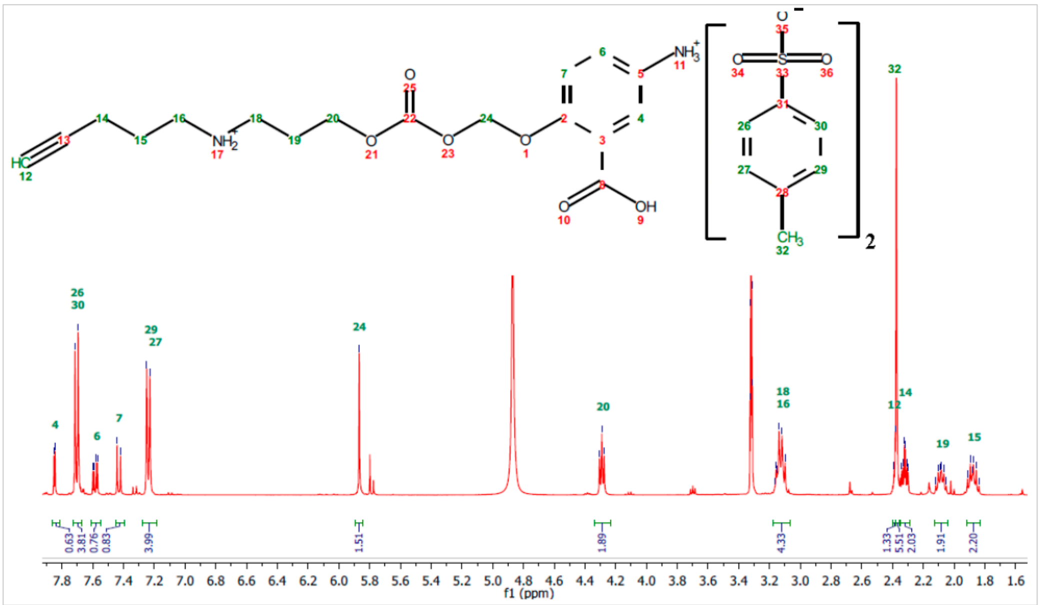

Figure S79. ^{1}H NMR spectrum of Msl prodrug **22** in MeOD.

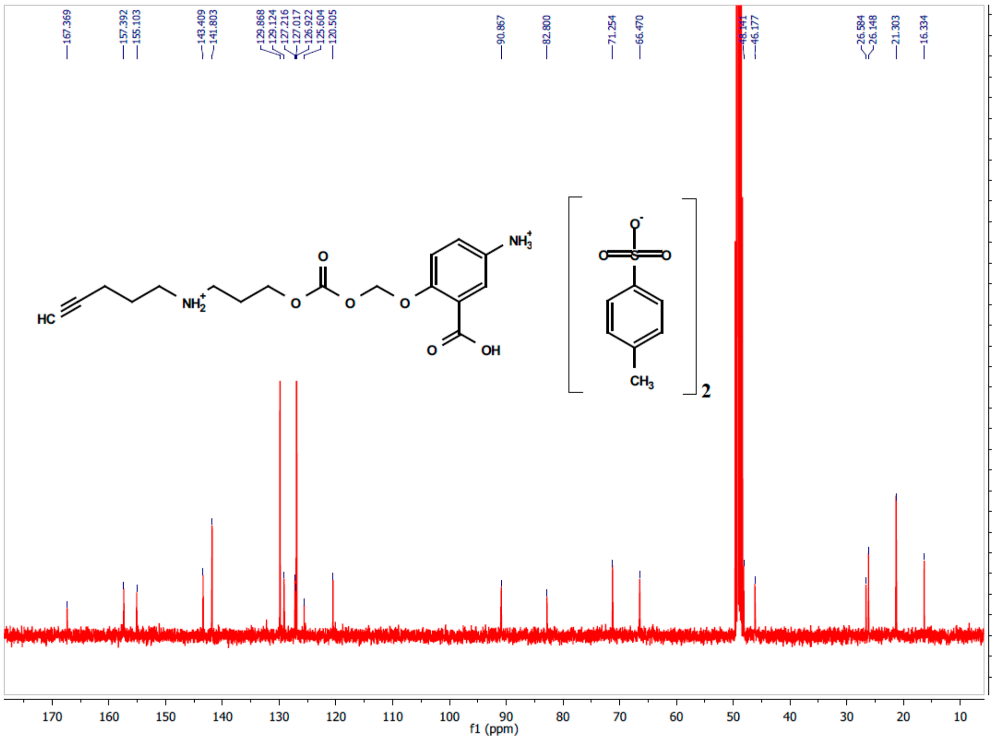

Figure S80. ^{13}C NMR spectrum of Msl prodrug **22** in MeOD.

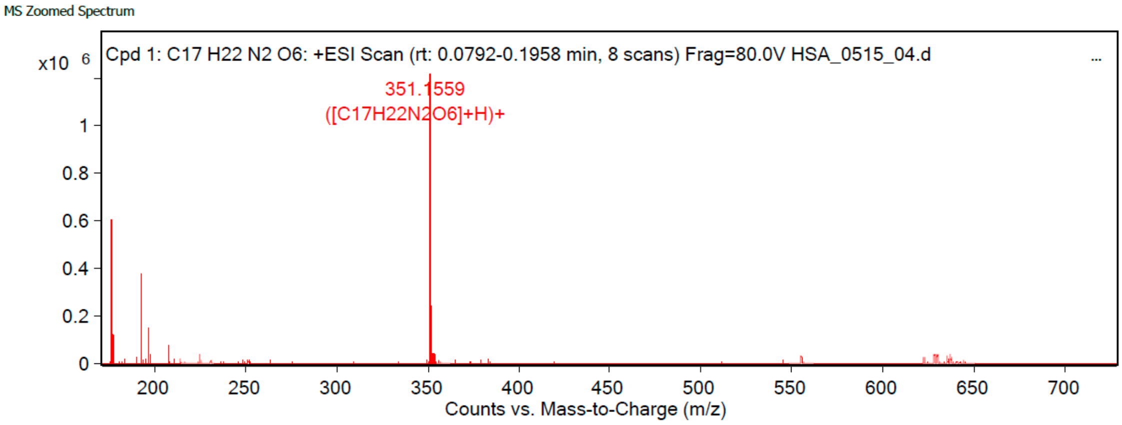

Figure S81. HRMS of Msl prodrug **22.**

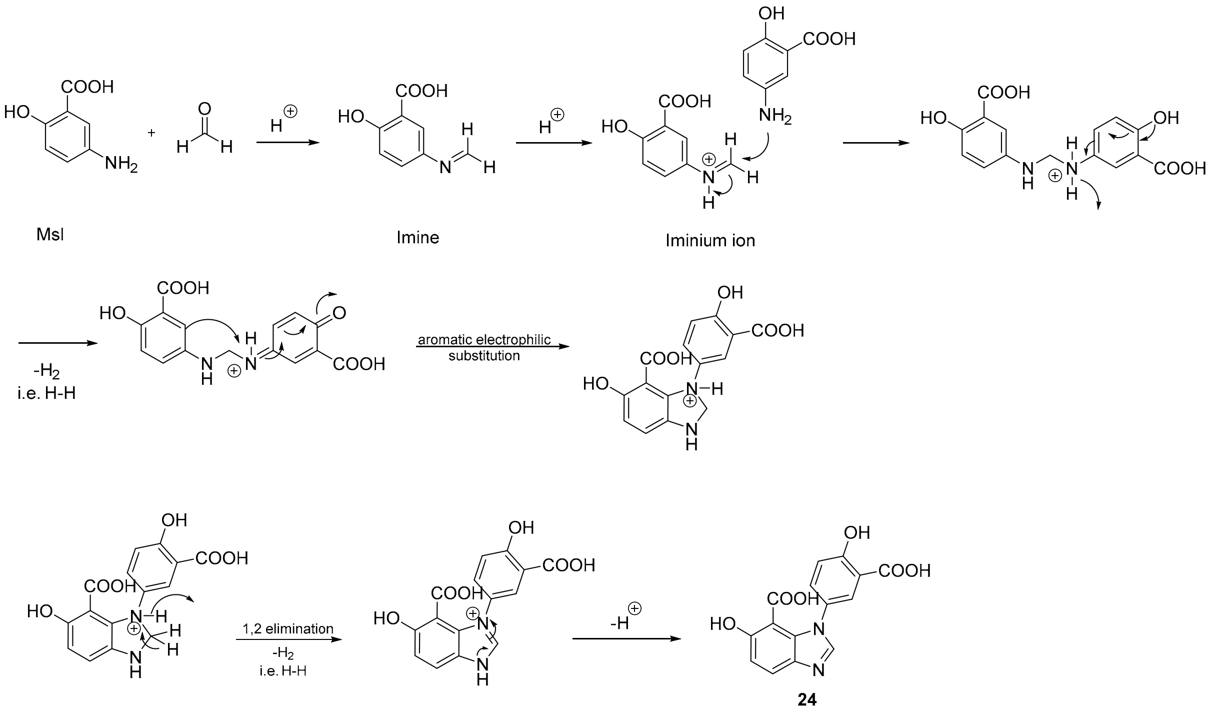

Scheme S3. Proposed mechanism for side-product **24** formation, which is supported by literature [46].
